# Supplementary material for: Incidence and risk factors of C. trachomatis and N. gonorrhoeae among young women from the Western Cape, South Africa: The EVRI study
Source: PLoS One. 2021 May 3;16(5):e0250871. doi: 10.1371/journal.pone.0250871 (PMC8092667; doi:10.1371/journal.pone.0250871)
Supplement: S1 File — (PDF) [file pone.0250871.s001.pdf]

Questions 1-4 to be filled in by the study coordinator:

1.Date of survey (day/month/year): |__|__| / |________________________/ |__|__|__|__|

2.PID #: | 7_|_0_|_ _|_ _|_ |

3.Date of birth (day/month/year): |__|__| /___________________________/ |__|__|__|__|

4.Study participant's initials: ___________

****

VISIT 1
STUDY QUESTIONNAIRE
THE EVRI STUDY

We appreciate your willingness to participate in this project.

All of the information you provide for us is strictly confidential, and your name will not be associated with this questionnaire and will never be used in reports.

Please provide the answer that best fits your situation.

5) Which one of the following would you say best represents your race? (Please check only one box)

- ☐ Asian
- ☐ Black
- ☐ Colored
- ☐ White
- ☐ Other

6) What is your current marital status? (Please check only one box)

- ☐ Single, never married
- ☐ Married
- ☐ Living together
- ☐ Divorced/Separated
- ☐ Widowed

7) How many years of school have you completed? (Please check only one box)

- [ ] I never attended school
- [ ] I did not finish standard 5
- [ ] I am currently a grade 1-7 learner
- [ ] I left school before completing grade 7
- [ ] I am currently a grade 8-12 learner
- [ ] I left school and did not pass grade 12
- [ ] I have passed matric/grade 12 but did not attend college/technikon/university
- [ ] I did attend some college/technikon/university before
- [ ] I am currently a student at college/technikon/university
- [ ] I have obtained a degree/diploma from college/technikon/university

8) Have you had at least one drink (beer, wine, or other drink with alcohol in it) in the past month?

- [ ] Yes
- [ ] No (Go to question 14)

9) During the past 1 month, on how many days did you have at least one drink with alcohol in it? (Please check only one box)

- [ ] One day
- [ ] Between 2-5 days
- [ ] More than 5 Days

10) On the days when you drank, about how many bottles of beer did you drink on average? (Enter 0 if none)

[ ]

11) On the days when you drank, about how many glasses of wine did you drink on average? (Enter 0 if none)

[ ]

12) On the days when you drank, about how many cocktails did you drink on average? (Enter 0 if none)

☐

13) On the days when you drank, about how many shots of liquor did you drink on average? (Enter 0 if none)

☐

14) Have you ever used any form of tobacco (cigarettes, roll-ups, pipes, cigars, chew, snuff)?

☐ Yes

☐ No (Go to introduction to question 23)

15) During your entire life, have you smoked at least 100 cigarettes/roll-ups, which is about 5 packs of cigarettes?

☐ Yes

☐ No (Go to question 21)

16) How old were you when you started smoking cigarettes/roll-ups?

☐ Years old

17) Since you started smoking, for how many years did you carry on smoking?

☐ Years

18) Do you smoke cigarettes/roll-ups now?

☐ Yes

☐ No (Go to question 20)

19) How many cigarettes/roll-ups do you smoke per day?

☐ Number of cigarettes/roll-ups (Go to question 21)

20) When you smoked in the past, how many cigarettes/roll-ups on average did you smoke per day?

☐ Cigarettes/roll-ups

21) Do you currently use chewing tobacco or snuff? (Please check only one box)

- [ ] Every Day
- [ ] Some Days
- [ ] Not at all

The following section will ask you questions about your reproductive health.

23) At what age did you start having your periods?

- [ ] Years of age

24) Have you ever used birth control?

- [ ] Yes
- [ ] No (Go to question 28)

25) What type of birth control method have you **ever** used? (*Check all that apply*)

- [ ] Oral contraceptive ("the pill")
- [ ] Diaphragm
- [ ] IUD/The Loop/Coil
- [ ] Condoms
- [ ] Foam, cream, jelly, suppositories
- [ ] Depo Provera or other injectable contraceptives
- [ ] Rhythm method
- [ ] Withdrawal
- [ ] Sterilization (tubes tied)
- [ ] Vasectomy (male partner sterilized)
- [ ] Other:___________________________________

26) Are you currently using birth control?

- [ ] Yes
- [ ] No (Go to questions 28)

27) What type of birth control method are you using **now**? (*Check all that apply*)

- ☐ Oral contraceptive (“the pill”)
- ☐ Diaphragm
- ☐ IUD/The Loop/Coil
- ☐ Condoms
- ☐ Foam, cream, jelly, suppositories
- ☐ Depo Provera or other injectable contraceptives
- ☐ Rhythm method
- ☐ Withdrawal
- ☐ Sterilization (tubes tied)
- ☐ Vasectomy (male partner sterilized)
- ☐ Other:___________________________________

28) Have you ever been pregnant?

- ☐ Yes
- ☐ No (Go to introduction to question 35)

29) How many times have you been pregnant? (Please fill in the number of times)

☐ Times

30) How many times have you had a child who was born normally (either vaginal or C-section) and was alive at birth ? (Enter 0 if none)

☐ Times

31) How many times have you had a termination of pregnancy (TOP)? (Enter 0 if none)

☐ Times

32) How many times have you had miscarriages (in the first 6 months of pregnancy)? (Enter 0 if none)

☐ Times

33) How many times have you had a baby born in the last 3 months of pregnancy that was not alive? (Enter 0 if none)

☐ Times

The next questions we are going to ask you are sensitive.

35) Has a doctor or health care provider ever diagnosed you with a sexually transmitted disease or infection (STD)?

- ☐ Yes
- ☐ No (Go to question 42)
- ☐ Don't know

36) Has a doctor or health care provider ever diagnosed you with genital warts?

- ☐ Yes
- ☐ No
- ☐ Don't know

37) Has a doctor or health care provider ever diagnosed you with genital herpes?

- ☐ Yes
- ☐ No
- ☐ Don't know

38) Has a doctor or health care provider ever diagnosed you with chlamydia?

- ☐ Yes
- ☐ No
- ☐ Don't know

39) Has a doctor or health care provider ever diagnosed you with gonorrhea?

- ☐ Yes
- ☐ No
- ☐ Don't know

40) Has a doctor or health care provider ever diagnosed you with syphilis (vuilsiek)?

- [ ] Yes
- [ ] No
- [ ] Don't' know

41) Has a doctor or health care provider ever diagnosed you with NGU (non-gonococcal urethritis)?

- [ ] Yes
- [ ] No
- [ ] Don't know

42) Has a doctor or health care provider ever diagnosed you with Hepatitis B?

- [ ] Yes
- [ ] No
- [ ] Don't know

43) Has a doctor or health care provider ever diagnosed you with Hepatitis C?

- [ ] Yes
- [ ] No
- [ ] Don't know

44) Has a doctor or health care provider ever diagnosed you with HIV?

- [ ] Yes
- [ ] No
- [ ] Don't know

45) Have you ever had a sex partner where you knew or thought that he had sexually transmitted disease (STD), or where you found out afterwards that he had a sexually transmitted disease (STD)?

- [ ] Yes
- [ ] No

46) Have you ever had a sex partner who had HIV, or where you found out afterwards that he had HIV?

- [ ] Yes
- [ ] No

47) Have you ever had a sex partner who had genital warts?

- [ ] Yes
- [ ] No
- [ ] Don't know

48) Have you ever had a male sex partner who was circumcised? (Please check only one box)

- [ ] Yes
- [ ] No
- [ ] Don't know

49) Is your regular male sex partner circumcised? (Please check only one box)

- [ ] Yes
- [ ] No
- [ ] Don't know
- [ ] Do not currently have a regular male sex partner

50) Have you ever had a Pap smear? (Please check only one box)

- [ ] Yes
- [ ] No (Go to introduction to question 54)
- [ ] Don't know (Go to introduction to question 54)

51) How old were you when you had your first Pap smear?

☐ Years

52) Have you ever had an abnormal Pap smear? (there was something wrong on the Pap smear). (Please check only one box)

☐ Yes

☐ No

☐ Don't know

The following section will ask you questions about sexual relations.

54) Have you ever had <u>vaginal sex</u>?

☐ Yes

☐ No (Go to question 65)

55) How old were you when you first had <u>vaginal sex</u>?

☐ Years old

56) In your life, what is the number of men with whom you have had <u>vaginal sex</u>?

☐ Men

57) Have you had vaginal sex <u>in the last six (6) months</u>?

☐ Yes

☐ No (Go to question 64)

58) <u>In the past 6 months</u>, how many different men have you had <u>vaginal sex</u> with?

☐ Men

59) <u>In the past 6 months</u>, how many men have you had <u>vaginal sex</u> with for the first time?

☐ Men

60) In the past 6 months, on average how often did you have vaginal sex? (Choose only one response)

- [ ] Less than once per month (Go to question 63)
- [ ] More than once a month (Go to question 62)
- [ ] More than once a week (Go to question 61)

61) In the past 6 months, on average how many times per week did you have vaginal sex?

[ ] Times per week (Go to question 63)

62) In the past 6 months, on average how many times per month did you have vaginal sex?

[ ] Times per month

63) In the past 6 months, when you had vaginal sex, how often did you or your partner use condoms? (Please check only one box)

- [ ] Always
- [ ] More than half the time
- [ ] Half the time
- [ ] Less than half the time
- [ ] Never

64) Did you or your partner use condoms the last time you had vaginal sex? (Please check only one box)

- [ ] Yes
- [ ] No
- [ ] Don’t remember
- [ ] Never used a condom with vaginal sex

65) Have you ever performed oral sex on a man?

☐ Yes

☐ No (Go to question 70)

66) Did you perform oral sex on a man in the past 6 months?

☐ Yes

☐ No (Go to question 70)

67) In the past 6 months, on average how often did you perform oral sex on a man?
(Choose only one response)

☐ Less than once a month (Go to question 70)

☐ More than once a month (Go to question 69)

☐ More than once a week (Go to question 68)

68) In the past 6 months, on average how many times per week did you perform oral sex on a man?

☐ Times (Go to question 70)

69) In the past 6 months, on average how many times per month did you perform oral sex on a man?

☐ Times

70) Has a man ever performed oral sex on you?

☐ Yes

☐ No (Go to question 75)

71) Did a man perform oral sex on you in the past 6 months?

☐ Yes

☐ No (Go to question 75)

72) In the past 6 months, on average how often did a man perform oral sex on you?
(Choose only one response)

☐ Less than once a month (Go to question 75)

☐ More than once a month (Go to question 74)

☐ More than once a week (Go to question 73)

73) In the past 6 months, how many times per week did a man perform oral sex on you?

☐ Times (Go to question 75)

74) In the past 6 months, how many times per month did a man perform oral sex on you?

☐ Times

75) Have you ever had anal sex?

☐ Yes

☐ No (Go to question 82)

76) Did you have anal sex in the past 6 months?

☐ Yes

☐ No (Go to question 81)

77) In the past 6 months, on average how often did you have anal sex?
(Choose only one response

☐ Less than once a month (Go to question 80)

☐ More than once a month (Go to question 79)

☐ More than once a week (Go to question 78)

78) In the past 6 months, on average how many times per week did you have anal sex?

☐ Times (Go to question 80)

79) In the past 6 months, on average how many times per month did you have anal sex?

☐ Times

80) In the past 6 months, when you had anal sex, how often did your partner use condoms? (Please check only one box)

☐ Always

☐ More than half the time

☐ Half the time

☐ Less than half the time

☐ Never

81) Did your partner use condoms the last time you had anal sex? (Please check only one box)

☐ Yes

☐ No

82) Have you ever received presents, money, or drugs from a man in exchange for sex?

☐ Yes

☐ No (Thank you for your time – please end the survey)

83) In the past 6 months, has a man given you presents, money, or drugs in exchange for sex?

☐ Yes

☐ No (Thank you for your time – please end the survey)

84) In the past 6 months, how many times has a man given you presents, money, or drugs in exchange for sex with you?

☐ Times

85) In the past 6 months, when you were given presents, money, or drugs for sex, how often did you or your partner use condoms? (Please check only one box)

- ☐ Always
- ☐ More than half the time
- ☐ Half the time
- ☐ Less than half the time
- ☐ Never

THANK YOU FOR YOUR TIME.
YOUR CONTRIBUTION IS VERY IMPORTANT TO OUR STUDY.
YOU ARE HELPING US TO PLAN FOR BETTER HEALTH CARE IN THE COMMUNITY.
