# Supplementary material for: Incidence and risk factors of C. trachomatis and N. gonorrhoeae among young women from the Western Cape, South Africa: The EVRI study
Source: PLoS One. 2021 May 3;16(5):e0250871. doi: 10.1371/journal.pone.0250871 (PMC8092667; doi:10.1371/journal.pone.0250871)
Supplement: S2 File — (PDF) [file pone.0250871.s002.pdf]

**Die studiekoördineerder moet vraag 1–4 invul.**

1. Datum van opname (dag/maand/jaar): |__|__| / ______________________ / |__|__|__|__|

2. PID #: | 7_|_0_|_ _|_ _|_ |

3. Geboortedatum (dag/maand/jaar): |__|__| / ________________________ / |__|__|__|__|

4. Voorletters van deelnemer aan studie: __________

**********************************************************************************************************

# BESOEK 1

# STUDIEVRAELYS
# DIE EVRI-STUDIE

Ons waardeer dit dat jy bereid is om aan hierdie projek deel te neem.

Alle inligting wat jy aan ons verskaf, sal streng vertroulik wees. Jou naam sal nie met jou vraelys verbind word nie, en sal nooit in verslae genoem word nie.

**Gee asseblief by elke vraag die antwoord wat jou situasie die beste beskryf.**

5) Watter een van die opsies hier onder beskryf jou ras die beste, sou jy sê? (Merk asseblief net een blokkie.)

☐ Asiaat

☐ Swart

☐ Bruin

☐ Wit

☐ Ander

6) Wat is jou huidige huwelikstaat? (Merk asseblief net een blokkie.)

☐ Enkellopend, en was nog nooit getroud nie

☐ Getroud

☐ In ’n saamwoonverhouding

☐ Geskei of uitmekaar

☐ Wewenaar/weduwee

7) Hoeveel jaar van skoolonderrig het jy voltooi? (Merk asseblief net een blokkie.)

- ☐ Ek het geen skoolopleiding nie.
- ☐ Ek nie standerd 5 voltooi nie.
- ☐ Ek is op die oomblik ’n leerder in graad 1–7.
- ☐ Ek het uit die skool gegaan voordat ek graad 7 voltooi het.
- ☐ Ek is op die oomblik ’n leerder in graad 8–12.
- ☐ Ek het uit die skool gegaan voordat ek graad 12 voltooi het.
- ☐ Ek het matriek/graad 12 deurgekom, maar nie kollege/tegnikon/universiteit toe gegaan nie.
- ☐ Ek het ’n ruk aan ’n kollege/tegnikon/universiteit gestudeer.
- ☐ Ek studeer op die oomblik aan ’n kollege/tegnikon/universiteit.
- ☐ Ek het ’n graad/diploma aan ’n kollege/tegnikon/universiteit verwerf.

8) Het jy in die afgelope maand minstens een drankie (bier, wyn of ander drank wat alkohol bevat) gehad?

- ☐ Ja
- ☐ Nee (Gaan na vraag 14.)

9) Hoeveel dae in <u>die afgelope maand</u> het jy minstens een drankie gehad wat alkohol bevat? (Merk asseblief net een blokkie.)

- ☐ Een dag
- ☐ Tussen 2 en 5 dae
- ☐ Meer as 5 dae

10) Ongeveer hoeveel bottels bier het jy gemiddeld gedrink op dié dae wanneer jy iets gedrink het? (Indien geen, skryf 0.)

☐

11) Ongeveer hoeveel glase wyn het jy gemiddeld gedrink op dié dae wanneer jy iets gedrink het? (Indien geen, skryf 0.)

☐

12) Ongeveer hoeveel mengeldrankies het jy gemiddeld gedrink op dié dae wanneer jy iets gedrink het? (Indien geen, skryf 0.)

☐

13) Ongeveer hoeveel sopies sterk drank het jy gemiddeld gedrink op dié dae wanneer jy iets gedrink het? (Indien geen, skryf 0.)

☐

14) Het jy al ooit enige tabakproduk gebruik (soos sigarette, selfgerolde sigarette, 'n pyp, sigare, pruimtabak, snuif)?

☐ Ja

☐ Nee (Gaan na die inleiding tot vraag 23.)

15) Het jy al in jou lewe minstens 100 sigarette/selfgerolde sigarette (dit is ongeveer 5 pakkies sigarette) gerook?

☐ Ja

☐ Nee (Gaan na vraag 21.)

16) Hoe oud was jy toe jy begin het om sigarette/selfgerolde sigarette te rook?

☐ jaar

17) Nadat jy begin rook het, hoeveel jaar het jy aanhou rook?

☐ jaar

18) Rook jy op die oomblik sigarette/selfgerolde sigarette?

☐ Ja

☐ Nee (Gaan na vraag 20.)

19) Hoeveel sigarette/selfgerolde sigarette rook jy per dag?

☐ Aantal sigarette/selfgerolde sigarette (Gaan na vraag 21.)

20) Toe jy nog gerook het, hoeveel sigarette/selfgerolde sigarette het jy gemiddeld op 'n dag gerook?

☐ Aantal sigarette/selfgerolde sigarette

21) Pruim of snuif jy op die oomblik tabak? (Merk asseblief net een blokkie.)

- ☐ Elke dag
- ☐ Party dae
- ☐ Glad nie

**Die volgende afdeling bevat vrae oor jou seksuele gesondheid.**

23) Op watter ouderdom het jy jou maandstonde begin kry?

- ☐ jaar

24) Het jy al ooit voorbehoedmiddels gebruik?

- ☐ Ja
- ☐ Nee (Gaan na vraag 28.)

25) Watter soort voorbehoeding het jy **al ooit** gebruik? (Merk alle blokkies wat van toepassing is.)

- ☐ Mondelikse voorbehoedmiddel (‘die pil’)
- ☐ Diafragma
- ☐ IUA/‘lussie’/‘veertjie’
- ☐ Kondome
- ☐ Skuim, room, jel, setpille
- ☐ Depo Provera of ander voorbehoedmiddels wat ingespuit word
- ☐ Ritmemetode
- ☐ Onttrekking
- ☐ Sterilisasie (buise afgebind)
- ☐ Vasektomie (manlike maat gesteriliseer)
- ☐ Ander: ___________________________________

26) Gebruik jy op die oomblik ’n voorbehoedmiddel?

- ☐ Ja
- ☐ Nee (Gaan na vraag 28.)

27) Watter soort voorbehoedmiddel gebruik jy **op die oomblik**? *(Merk alle blokkies wat van toepassing is.)*

- [ ] Mondelikse voorbehoedmiddel ('die pil')
- [ ] Diafragma
- [ ] IUA/'lussie'/'veertjie'
- [ ] Kondome
- [ ] Skuim, room, jel, setpille
- [ ] Depo Provera of ander voorbehoedmiddels wat ingespuit word
- [ ] Ritmemetode
- [ ] Onttrekking
- [ ] Sterilisasie (buise afgebind)
- [ ] Vasektomie (manlike maat gesteriliseer)
- [ ] Ander: ___________________________________

28) Was jy al ooit swanger?

- [ ] Ja
- [ ] Nee (Gaan na die inleiding tot vraag 35.)

29) Hoeveel keer was jy al swanger? (Skryf asseblief die aantal kere neer.)

[ ] keer

30) Hoeveel keer het jy normaal geboorte geskenk (vaginaal óf met 'n keisersnit) en het die kind by geboorte geleef? (Indien geen, skryf 0.)

[ ] keer

31) Hoeveel keer het jy al 'n beëindiging van swangerskap (BVS) gehad? (Indien geen, skryf 0.)

[ ] keer

32) Hoeveel keer het jy al 'n miskraam gehad (binne die eerste 6 maande van 'n swangerskap)? (Indien geen, skryf 0.)

[ ] keer

33) Hoeveel keer het jy 'n baba gekry wat gedurende die laaste 3 maande van 'n swangerskap doodgebore is? (Indien geen, skryf 0.)

[ ] keer

**Die vrae wat nou volg, gaan oor sensitiewe sake.**

35) Het ’n dokter of gesondheidsorgwerker jou al ooit met ’n seksueel oordraagbare siekte of infeksie (SOS) gediagnoseer?

- [ ] Ja
- [ ] Nee (Gaan na vraag 42.)
- [ ] Weet nie

36) Het ’n dokter of gesondheidsorgwerker jou al ooit met vratte op die geslagsdele gediagnoseer?

- [ ] Ja
- [ ] Nee
- [ ] Weet nie

37) Het ’n dokter of gesondheidsorgwerker jou al ooit met herpes op die geslagsdele gediagnoseer?

- [ ] Ja
- [ ] Nee
- [ ] Weet nie

38) Het ’n dokter of gesondheidsorgwerker jou al ooit met *Chlamydia*-infeksie gediagnoseer?

- [ ] Ja
- [ ] Nee
- [ ] Weet nie

39) Het ’n dokter of gesondheidsorgwerker jou al ooit met gonorree gediagnoseer?

- [ ] Ja
- [ ] Nee
- [ ] Weet nie

40) Het ’n dokter of gesondheidsorgwerker jou al ooit met sifilis (‘vuilsiek’) gediagnoseer?

- [ ] Ja
- [ ] Nee
- [ ] Weet nie

41) Het ’n dokter of gesondheidsorgwerker jou al ooit met NGU (nie-gonokokkale uretritis) gediagnoseer?

- [ ] Ja
- [ ] Nee
- [ ] Weet nie

42) Het ’n dokter of gesondheidsorgwerker jou al ooit met Hepatitis B gediagnoseer?

- [ ] Ja
- [ ] Nee
- [ ] Weet nie

43) Het ’n dokter of gesondheidsorgwerker jou al ooit met Hepatitis C gediagnoseer?

- [ ] Ja
- [ ] Nee
- [ ] Weet nie

44) Het ’n dokter of gesondheidsorgwerker jou al ooit met MIV gediagnoseer?

- [ ] Ja
- [ ] Nee
- [ ] Weet nie

45) Het jy ooit seks gehad met iemand van wie jy geweet of vermoed het dat die persoon ’n seksueel oordraagbare siekte (SOS) het, of van wie jy agterna uitgevind het dat die persoon ’n SOS het?

☐ Ja

☐ Nee

46) Het jy ooit seks gehad met iemand wat MIV het, of van wie jy agterna uitgevind het dat die persoon MIV het?

☐ Ja

☐ Nee

47) Het jy al ooit seks gehad met iemand wat vratte op sy of haar geslagsdele het?

☐ Ja

☐ Nee

☐ Weet nie

48) Het jy al ooit seks gehad met ’n man wat besny is? (Merk asseblief net een blokkie.)

☐ Ja

☐ Nee

☐ Weet nie

49) Is die man met wie jy gereeld seks het, besny? (Merk asseblief net een blokkie.)

☐ Ja

☐ Nee

☐ Weet nie

☐ Ek het op die oomblik met geen man gereeld seks nie

50) Het jy al ooit ’n Pap-smeer gehad? (Merk asseblief net een blokkie.)

☐ Ja

☐ Nee (Gaan na die inleiding tot vraag 54.)

☐ Weet nie (Gaan na die inleiding tot vraag 54.)

51) Hoe oud was jy toe jy die eerste keer ’n Pap-smeer gehad het?

☐ jaar

52) Het jou Pap-smeer al ooit ’n abnormale uitslag gehad? (Dit wil sê, die toets het gewys iets is verkeerd.) (Merk asseblief net een blokkie.)

☐ Ja

☐ Nee

☐ Weet nie

**Die volgende afdeling bevat vrae oor jou seksuele verhoudings.**

54) Het jy al ooit vaginale seks gehad?

☐ Ja

☐ Nee (Gaan na vraag 65.)

55) Hoe oud was jy toe jy die eerste keer vaginale seks gehad het?

☐ jaar

56) Met hoeveel mans het jy al altesaam in jou lewe vaginale seks gehad?

☐ mans

57) Het jy in die afgelope 6 maande vaginale seks gehad?

☐ Ja

☐ Nee (Gaan na vraag 64.)

58) Met hoeveel mans het jy die afgelope 6 maande vaginale seks gehad?

☐ mans

59) Met hoeveel mans het jy die afgelope 6 maande vir die eerste keer vaginale seks gehad?

☐ mans

60) Ongeveer hoe dikwels het jy die afgelope 6 maande vaginale seks gehad? (Merk asseblief net een blokkie.)

- [ ] Minder as een keer ’n maand (Gaan na vraag 63.)
- [ ] Meer as een keer ’n maand (Gaan na vraag 62.)
- [ ] Meer as een keer ’n week (Gaan na vraag 61.)

61) Ongeveer hoeveel keer ’n week het jy die afgelope 6 maande vaginale seks gehad?

[ ] keer per week (Gaan na vraag 63.)

62) Ongeveer hoeveel keer ’n maand het jy die afgelope 6 maande vaginale seks gehad?

[ ] keer ’n maand

63) Hoe gereeld het julle ’n kondoom gebruik wanneer jy die afgelope 6 maande vaginale seks gehad het? (Merk asseblief net een blokkie.)

- [ ] Altyd
- [ ] Meer as die helfte van die kere
- [ ] Die helfte van die kere
- [ ] Minder as die helfte van die kere
- [ ] Nooit

64) Het julle ’n kondoom gebruik die laaste keer toe jy vaginale seks gehad het? (Merk asseblief net een blokkie.)

- [ ] Ja
- [ ] Nee
- [ ] Kan nie onthou nie
- [ ] Het nog nooit ’n kondoom tydens vaginale seks gebruik nie

65) Het jy al ooit vir ’n man orale seks gegee?

- [ ] Ja
- [ ] Nee (Gaan na vraag 70.)

66) Het jy in die afgelope 6 maande vir ’n man orale seks gegee?

- [ ] Ja
- [ ] Nee (Gaan na vraag 70.)

67) Ongeveer hoe dikwels het jy die afgelope 6 maande vir ’n man orale seks gegee? (Merk asseblief net een blokkie.)

- [ ] Minder as een keer ’n maand (Gaan na vraag 70.)
- [ ] Meer as een keer ’n maand (Gaan na vraag 69.)
- [ ] Meer as een keer ’n week (Gaan na vraag 68.)

68) Ongeveer hoeveel keer ’n week het jy die afgelope 6 maande vir ’n man orale seks gegee?

- [ ] keer (Gaan na vraag 70.)

69) Ongeveer hoeveel keer ’n maand het jy die afgelope 6 maande vir ’n man orale seks gegee?

- [ ] keer

70) Het ’n man jou al ooit orale seks gegee?

- [ ] Ja
- [ ] Nee (Gaan na vraag 75.)

71) Het ’n man jou die afgelope 6 maande orale seks gegee?

- [ ] Ja
- [ ] Nee (Gaan na vraag 75.)

72) Ongeveer hoe dikwels het ’n man jou die afgelope 6 maande orale seks gegee?
(Merk asseblief net een blokkie.)

- [ ] Minder as een keer ’n maand (Gaan na vraag 75.)
- [ ] Meer as een keer ’n maand (Gaan na vraag 74.)
- [ ] Meer as een keer ’n week (Gaan na vraag 73.)

73) Ongeveer hoeveel keer ’n week het ’n man jou die afgelope 6 maande orale seks gegee?

- [ ] keer (Gaan na vraag 75.)

74) Ongeveer hoeveel keer ’n maand het ’n man jou die afgelope 6 maande orale seks gegee?

- [ ] keer

75) Het jy al ooit anale seks gehad?

- [ ] Ja
- [ ] Nee (Gaan na vraag 82.)

76) Het jy in die afgelope 6 maande anale seks gehad?

- [ ] Ja
- [ ] Nee (Gaan na vraag 81.)

77) Ongeveer hoe dikwels het jy die afgelope 6 maande anale seks gehad?
(Merk asseblief net een blokkie.)

- [ ] Minder as een keer ’n maand (Gaan na vraag 80.)
- [ ] Meer as een keer ’n maand (Gaan na vraag 79.)
- [ ] Meer as een keer ’n week (Gaan na vraag 78.)

78) Ongeveer hoeveel keer ’n week het jy die afgelope 6 maande anale seks gehad?

- [ ] keer (Gaan na vraag 80.)

79) Ongeveer hoeveel keer ’n maand het jy <u>die afgelope 6 maande anale seks</u> gehad?

☐ keer

80) Hoe gereeld het julle kondome gebruik wanneer jy <u>die afgelope 6 maande anale seks</u> gehad het? (Merk asseblief net een blokkie.)

☐ Altyd
☐ Meer as die helfte van die kere
☐ Die helfte van die kere
☐ Minder as die helfte van die kere
☐ Nooit

81) Het jou seksmaat ’n kondoom gebruik die laaste keer toe julle <u>anale seks</u> gehad het? (Merk asseblief net een blokkie.)

☐ Ja
☐ Nee

82) Het ’n man jou al ooit geskenke, geld of dwelmmiddels gegee in ruil daarvoor om seks met jou te hê?

☐ Ja
☐ Nee (Dankie vir jou tyd. Jy kan die vraelys hier beëindig.)

83) Het ’n man jou <u>die afgelope 6 maande</u> geskenke, geld of dwelmmiddels in ruil vir seks gegee?

☐ Ja
☐ Nee (Dankie vir jou tyd. Jy kan die vraelys hier beëindig.)

84) Hoeveel keer het ’n man jou die afgelope 6 maande geskenke, geld of dwelmmiddels in ruil vir seks gegee?

☐ keer

85) Hoe gereeld het julle 'n kondoom gebruik wanneer jy die afgelope 6 maande geskenke, geld of dwelmmiddels in ruil vir seks gekry het? (Merk asseblief net een blokkie.)

- [ ] Altyd
- [ ] Meer as die helfte van die kere
- [ ] Die helfte van die kere
- [ ] Minder as die helfte van die kere
- [ ] Nooit

**DANKIE VIR JOU TYD.**
**JOU BYDRAE IS BAIE BELANGRIK VIR ONS STUDIE.**
**JY HELP ONS OM VIR BETER GESONDHEIDSORG IN DIE GEMEENSKAP TE BEPLAN.**
