# Supplementary material for: Incidence and risk factors of C. trachomatis and N. gonorrhoeae among young women from the Western Cape, South Africa: The EVRI study
Source: PLoS One. 2021 May 3;16(5):e0250871. doi: 10.1371/journal.pone.0250871 (PMC8092667; doi:10.1371/journal.pone.0250871)
Supplement: S3 File — (PDF) [file pone.0250871.s003.pdf]

Umbuzo woku-1-4 kufuneka uzaliswe ngumququzeleli wesi sifundo:

1.Umhla wodliwano-ndlebe (umhla/inyanga/unyaka): |__|__| / ____________/ |__|__|__|__|

2.IPID #: | 7_|_0_|_ _|_ _|_ |

3.Umhla wokuzalwa (umhla/inyanga/unyaka): |__|__| / _________________/ |__|__|__|__|

4.Oonobumba bokuqala begama lalowo uthatha inxaxheba kwisifundo: _________

****

UTYELELO LOKU-1

IPHEPHA ELINEMIBUZO NESIFUNDO
ISIFUNDO SE-EVRI

Siyakuvuyela ukuba nomdla kwakho ukuthatha inxaxheba kule projekthi.

Zonke iinkcukacha osinika zona ziza kuba yimfihlelo, kwaye igama lakho alizi kunxulunyaniswa nawe kwaye soze lisetyenziswe kwiingxelo.

Nceda usinike impendulo elungelelana nemeko yakho.

5) Yeyiphi kwezi zilandelayo onokuthi imele uhlanga lwakho? (Nceda uphawule ibhokisi ibenye)

- ☐ Um-Asia
- ☐ OMnyama
- ☐ OweBala
- ☐ OMhlophe
- ☐ Olunye

6) Sithini isimo sakho somtshato? (Nceda uphawule ibhokisi ibenye)

- ☐ Awutshatanga, zange utshate
- ☐ Utshatile
- ☐ Nihlala kunye

☐ Niqhawule umsthato/nahlukene

☐ Ngumhlolokazi

7) Uphele kubani esikolweni? (Nceda uphawule ibhokisi ibenye)

☐ Zange ndiye esikolweni

☐ Khange ndiligqibe ibanga lesi-5

☐ Ngoku ndingumfundi okwibakala loku-1-7

☐ Ndisishiye isikolo phambi kokuba ndigqibe ibakala lesi-7

☐ Ngoku ndingumfundi okwibakala lesi-8-12

☐ Ndisishiye isikolo kwaye khange ndiliphumelele ibakala le-12

☐ Ndilipasile ibanga leshumi/ibakala le-12 kodwa khange ndiye ekholejini/eteknikhoni/eyunivesithi

☐ Ndiyile ngaphambili ekholejini/eteknikhoni/eyunivesithi

☐ Ngoku ndingumfundi wasekholejini/eteknikhoni/eyunivesithi

☐ Ndifumene isidanga/idiploma ekholejini/eteknikhoni/eyunivesithi

8) Ubukhe wasela ubuncinane isiselo esinye (sebhiya, sewayini, okanye esinye isiselo esinxilisayo) kwinyanga enye edlulileyo?

☐ Ewe

☐ Hayi (Yiya kumbuzo wesi-14)

9) Kwinyanga e-1 edlulileyo, zingaphi iintsuku obukhe wasela ngazo ubuncinane isiselo esinye esinxilisayo? (Nceda uphawule ibhokisi ibenye)

☐ Usuku olunye

☐ Phakathi kweentsuku ezi-2-5

☐ Ngaphezu kweentsuku ezi-5

10) Kwiintsuku osela ngazo, ubusela malunga nomlinganiselo weebhotile ezingaph zebhiya? (Faka u-0 ukuba akukho nanye)

☐

11) Kwiintsuku osela ngazo, ubusela malunga nomlinganiselo weeglasi zewayini ezingaphi? (Faka u-0 ukuba akukho nanye)

☐

12) Kwiintsuku osela ngazo, ubusela malunga nomlinganiselo wemixube yewayini engaphi? (Faka u-0 ukuba akukho nanye)

☐

13) Kwiintsuku osela ngazo, ubusela malunga nomlinganiselo weethothi ezingaphi zotywala? (Faka u-0 ukuba akukho nanye)

☐

14) Wakhe walusebenzisa naluphi na uhlobo lwecuba (isigarethi, imirolo, ipeyipu, ezihlafunwayo, izineyifu)?

☐ Ewe

☐ Hayi (Yiya kwintshayelelo yombuzo wama-23)

15) Kubomi bakho bubonke, wakhe watshaya ubuncinane izigarethi/imirolo eli-100, emalunga neepakethi ezi-5 zesigarethi?

☐ Ewe

☐ Hayi (Yiya kumbuzo wama-21)

16) Ubunangaphi ukuqala kwakho ukutshaya isigarethi/imirolo?

☐ Iminyaka ubudala

17) Ukususeka oko uqalile ukutshaya, uqhubeke utshaya iminyaka engaphi?

☐ Iminyaka

18) Uyayitshaya na ngoku isigarethi/imirolo?

☐ Ewe

☐ Hayi (Yiya kumbuzo wama-20)

19) Zingaphi iisigarethi/imirolo oyitshayayo ngosuku?

☐ Inani leesigarethi/imirolo (Yiya kumbuzo wama-21)

20) Ngoku ubutshaya ngaphambili, bezingaphi iisgarethi/imirolo ngokomlinganiselo ubuyitshaya ngosuku?

☐ Iisigarethi/imirolo

21) Ingaba ngoku usebenzisa icuba elihlafunwayo okanye isinifu? (Nceda ujonge ibhokisi enye kuphela)

☐ Yonke imihla

☐ Ngezinye iintsuku

☐ Zange

Eli candelo lilandelayo liza kukubuza imibuzo emalunga nempilo kwiimeko zakho zokuzala

23) Uqale unangaphi ukuba sexesheni?

☐ Iminyaka yobudala

24) Wakhe wazisebenzisa izinto zokucwangcisa inzala?

☐ Ewe

☐ Hayi (Yiya kumbuzo wama-28)

25) Loluphi uhlobo lokuthintela ukuzala owakhe wayisebenzisa? (Jonga konke okusebenzayo kuwe)

☐ Izinto ezityiwayo zokucwangcisa ("iipilisi")
☐ Isivalo
☐ I-IUD/Iluphu/Isixhobo esifakwa esibelekweni sokuthintela ukuzala
☐ Iikhondom
☐ Ugwebu, ikhrimu, ijeli, iyeza elifakwa ebufazini elinyibilikayo
☐ IDepo okanye ezinye izinto zokucwangcisa ezifana neenaliti
☐ Indlela yokuzibalela ukuba uya nini na exesheni
☐ Ukuyikhupha
☐ Ukuvala (ukubotshwa kweetyhubhu)
☐ Ukuvalwa kwenzala ebudodeni (ukuvala komlingane wakhe inzala umphelo)

☐ Olunye:___________________________________

26) Ingaba ngoku zikhona izinto ozisebenzisayo zokucwangcisa inzala?

☐ Ewe

☐ Hayi (Yiya kumbuzo wama-28)

27) Loluphi uhlobo lokuthintela ukuzala olusebenzisayo **ngoku**? (*Jonga konke okusebenzayo kuwe*)

☐ Izinto ezityiwayo zokucwangcisa (“iipilisi”)
☐ Isivalo
☐ I-IUD/Iluphu/Isixhobo esifakwa esibelekweni sokuthintela ukuzala
☐ Iikhondom
☐ Ugwebu, ikhrimu, ijeli, iyeza elifakwa ebufazini elinyibilikayo
☐ IDepo okanye ezinye izinto zokucwangcisa ezifana neenaliti
☐ Indlela yokuzibalela ukuba uya nini na exesheni
☐ Ukuyikhupha
☐ Ukuvala (ukubotshwa kweetyhubhu)
☐ Ukuvalwa kwenzala ebudodeni (ukuvala komlingane wakhe inzala umphelo)
☐ Olunye___________________________________

28) Wakhe wakhulelwa?

☐ Ewe

☐ Hayi (Yiya kwintshayelelo yombuzo wama-35)

29) Mangaphi amaxesha owakhe wakhulelwa ngawo? (Nceda ubhale inani lamaxesha)

☐ Amaxesha

30) Zingaphi izihlandlo owathi wabafumana abantwana ngendlela yesiqhelo (ngokubakhupha ngaphantsi okanye ngokusikwa) kwaye bephila xa ubazala? (Faka u-0 ukuba akukho nasinye)

☐ Izihlandlo

31) Zingaphi izihlandlo owakhe wakhutshwa ngazo isisu ukhulelwe (iTOP)? (Faka u-0 ukuba akukho nasinye)

☐ Izihlandlo

32) Zingaphi izihlandlo apho wakhe waphunyelwa zizisu (kwiinyanga ezi-6 zokuqala ukhulelwe? (Faka u-0 ukuba akukho nasinye)

☐ Izihlandlo

33) Zingaphi izihlandlo apho wakhe wafumana khona umntu kwiinyanga ezi-3 zokugqibela ukhulelwe engaphili? (Faka u-0 ukuba akukho nasinye)

☐ Izihlandlo

Umbuzo olandelayo esiza kukubuza yona ivakala buthathaka.

35) Wakhe ugqirha okanye unompilo wakufumanisa unesifo esosulela ngokwabelana ngesondo okanye into eyosulelayo (iiSTD)?

☐ Ewe

☐ Hayi (Yiya kumbuzo wama-42)

☐ Andazi

36) Ingaba ugqirha okanye unompilo wakhe wakufumanisa uneentsumpa ebufazini?

☐ Ewe

☐ Hayi

☐ Andazi

37) Ingaba ugqirha okanye unompilo wakhe wakufumanisa utshotshozela kusisi?

☐ Ewe

☐ Hayi

☐ Andazi

38) Ingaba ugqirha okanye unompilo wakhe wakufumanisa unesifo esosulelana ngenxa yeentsholongwane?

☐ Ewe

☐ Hayi

☐ Andazi

39) Ingaba ugqirha okanye unompilo wakhe wakufumanisa unegonoriya ubhobhozo?

- [ ] Ewe
- [ ] Hayi
- [ ] Andazi

40) Ingaba ugqirha okanye unompilo wakhe wakufumanisa unegcushuwa (ivuilsiek)?

- [ ] Ewe
- [ ] Hayi
- [ ] Andazi

41) Ingaba ugqirha okanye unompilo wakhe wakufumanisa uneNGU (ukudumba kwesinyi)?

- [ ] Ewe
- [ ] Hayi
- [ ] Andazi

42) Ingaba ugqirha okanye unompilo wakhe wakufumanisa udumbe isibindi ngenxa yokosuleleka?

- [ ] Ewe
- [ ] Hayi
- [ ] Andazi

43) Ingaba ugqirha okanye unompilo wakhe wakufumanisa unesifo esikhokelela ekudumbeni kwesibindi?

- [ ] Ewe
- [ ] Hayi
- [ ] Andazi

44) Ingaba ugqirha okanye unompilo wakhe wakufumanisa uneNtsholongwane kaGawulayo?

- [ ] Ewe
- [ ] Hayi
- [ ] Andazi

45) Wakhe wanalo iqabane owabelana nalo ngesondo apho wawusazi okanye ulicingela ukuba linesifo esosulela ngokwabelana ngesondo (iiSTD), okanye apho uye wafumaniusa mva ukuba ebenesifo esosulela ngokwabelana ngesondo (iSTD)?

- [ ] Ewe
- [ ] Hayi

46) Wakhe wanalo iqabane owabelana ngesondo elakhe laneNtsholongwane kaGawulayo, okanye apho wafumanisa kamva ukuba lineNtsholongwane kaGawulayo?

- [ ] Ewe
- [ ] Hayi

47) Wakhe wanalo iqabane owabelana nalo ngesondo elakhe lanesifo esosulela ngesondo?

- [ ] Ewe
- [ ] Hayi
- [ ] Andazi

48) Wakhe wanalo iqabane eliyindoda owabelana nalo ngesondo elakhe loluka? (Nceda ukhethe ibhokisi enye kuphela)

- [ ] Ewe
- [ ] Hayi
- [ ] Andazi

49) Ingaba iqabane lakho eliyindoda owabelana nalo ngesondo rhoqo lolukile? (Nceda ukhethe ibhokisi enye kuphela)

- [ ] Ewe
- [ ] Hayi

☐ Andazi

☐ Okwangoku andinalo iqabane eliyindoda endabelana nalo rhoqo ngesondo

50) Wakhe wacocwa isibeleko? (Nceda ukhethe ibhokisi enye kuphela)

☐ Ewe

☐ Hayi (Yiya kwintshayelelo yombuzo wama-54)

☐ Andazi (Yiya kwintshayelelo yombuzo wama-54)

51) Wawunangaphi ukucocwa kwakho okokuqala isibeleko?

☐ Iminyaka

52) Wakhe wacocwa isibeleko? (yayikhona into engahambi kakuhle ngoku wawucocwa isibeleko). (Nceda uphawule ibhokisi ibenye kuphela)

☐ Ewe

☐ Hayi

☐ Andazi

Eli candela lilandelayo liza kukubuza imibuzo ngezinxulumene nezesondo,

54) Wakhe wabelana ngesondo ngokulalana?

☐ Ewe

☐ Hayi (Yiya kumbuzo wama-65)

55) Wawunangaphi ukwabelana kwakho ngesondo ngokulalana?

☐ Iminyaka ubudala

56) Ebomini bakho, mangaphi amadoda okwakhe wabelana nawo ngesondo ngokulalana?

☐ Amadoda

57) Wakhe wabelana ngesondo ngokulalana kwiinyanga ezintandathu (ezi-6) ezidlulileyo?

☐ Ewe

☐ Hayi (Yiya kumbuzo wama-64)

58) Kwiinyanga ezi-6 ezidlulileyo, mangaphi amadoda ohluka-hlukeneyo owakhe wabelana ngesondo ngokulalana nawo?

☐ Amadoda

59) Kwiinyanga ezi-6 ezidlulileyo, mangaphi amadoda owakhe wabelana ngesondo ngkulalana nawo okokuqala?

☐ Amadoda

60) Kwiinyanga ezi-6 ezidlulileyo, ngokomlinganiselo mangaphi amadoda owakhe wabelana ngesondo ngokulalana nawo? (Nceda ukhethe impendulo ibenye kuphela)

☐ Ngaphantsi kwakanye ngenyanga (Yiya kumbuzo wama-63)

☐ Ngaphezu kwakanye ngenyanga (Yiya kumbuzo wama-62)

☐ Ngaphezu kwakanye ngeveki (Yiya kumbuzo wama-61)

61) Kwiinyanga ezi-6 ezidlulileyo, ngokomlinganiselo zingaphi izihlandlo ngeveki okhe wabelana ngesondo ngokulalana?

☐ Izihlandlo ngeveki (Yiya kumbuzo wama-63)

62) Kwiinyanga ezi-6 ezidlulileyo, ngokomlinganiselo zingaphi izihlandlo ngenyanga okhe wabelana ngesondo ngokulalana?

☐ Izihlandlo ngenyanga

63) Kwiinyanga ezi-6 ezidlulileyo, ngoku wawusabelana ngesondo ngokulalana, wean okanye iqabane lakhe naniyisebenzisa kangaphi ikhondom? (Nceda uphawule ibhokisi ibenye kuphela)

☐ Soloko

☐ Ngaphezu kwesiqingatha sexesha

☐ Isiqingatha sexesha

☐ Ngaphantsi kwesiqingatha sexesha

☐ Zange

64) Ingaba wena okanye iqabane lakho nakhe nasebenzisa iikhondom kwixesha elidlulileyo nisabelana ngesondo ngokulalana? (Nceda uphawule ibhokisi ibenye kuphela)

☐ Ewe

☐ Hayi

☐ Andazi

☐ Zange ndisebenzise khondom xa ndisabelana ngesondo ngokulalana

65) Wakhe wabelana ngesondo ngomlomo nendoda?

☐ Ewe

☐ Hayi (Yiya kumbuzo wama-70)

66) Wakhe wabelana ngesondo ngomlomo nendoda kwiinyanga ezi-6 ezidlulileyo?

☐ Ewe

☐ Hayi (Yiya kumbuzo wama-70)

67) Kwiinyanga ezi-6 ezidlulileyo, wakhe wabelana ngesondo ngomlomo? (Khetha impendulo ibenye kuphela)

☐ Ngaphantsi kwakanye ngenyanga (Yiya kumbuzo wama-70)

☐ Ngaphezu kwakanye ngenyanga (Yiya kumbuzo wama-69)

☐ Ngaphezu kwakanye ngeveki (Yiya kumbuzo wama-68)

68) Kwiinyanga ezi-6 ezidlulileyo, ngokomlinganiselo zingaphi izihlandlo owakhe wabelana ngesondo ngeveki ngomlomo nendoda?

☐ Izihlandlo (Yiya kumbuzo wama-70)

69) Kwiinyanga ezi-6 ezidlulileyo, ngokomlinganiselo zingaphi izihlandlo owakhe wabelana ngesondo ngenyanga ngomlomo nendoda?

☐ Izihlandlo

70) Ingaba yakhe indoda yabelana ngesondo ngomlomo nawe?

- [ ] Ewe
- [ ] Hayi (Yiya kumbuzo wama-75)

71) Ingaba yakhe indoda yabelana ngesondo ngomlomo nawe, kwiinyanga ezi-6 ezidlulileyo?

- [ ] Ewe
- [ ] Hayi (Yiya kumbuzo wama-75)

72) Kwiinyanga ezi-6 ezidlulileyo, ngokomlinganiselo mangaphi amaxesha usabelana ngesondo ngomlomo nawo? (Khetha impendulo ibenye kuphela)

- [ ] Ngaphantsi kwakanye ngenyanga (Yiya kumbuzo wama-75)
- [ ] Ngaphezu kwakanye ngenyanga (Yiya kumbuzo wama-74)
- [ ] Ngaphezu kwakanye ngeveki (Yiya kumbuzo wama-73)

73) Kwiinyanga ezi-6 ezidlulileyo, zingaphi izihlandlo owakhe wabelana ngesondo ngeveki ngomlomo nendoda?

- [ ] Izihlandlo (Yiya kumbuzo wama-75)

74) Kwiinyanga ezi-6 ezidlulileyo, ngokomlinganiselo zingaphi izihlandlo owakhe wabelana ngesondo ngenyanga ngomlomo nendoda?

- [ ] Izihlandlo

75) Wakhe wabelana ngesondo ngemva?

- [ ] Ewe
- [ ] Hayi (Yiya kumbuzo wama-82)

76) Wakhe wabelana ngesondo ngemva, kwiinyanga ezi-6 ezidlulileyo?

- [ ] Ewe
- [ ] Hayi (Yiya kumbuzo wama-81)

77) Kwiinyanga ezi-6 ezidlulileyo, ngokomlinganiselo mangaphi amaxesha usabelana ngesondo ngemva? (Khetha impendulo ibenye kuphela)

☐ Ngaphantsi kwakanye ngenyanga (Yiya kumbuzo wama-80)

☐ Ngaphezu kwakanye ngenyanga (Yiya kumbuzo wama-79)

☐ Ngaphezu kwakanye ngeveki (Yiya kumbuzo wama-78)

78) Kwiinyanga ezi-6 ezidlulileyo, ngokomlinganiselo zingaphi izihlandlo owakhe wabelana ngesondo ngeveki ngasemva?

☐ Izihlandlo (Yiya kumbuzo wama-80)

79) Kwiinyanga ezi-6 ezidlulileyo, ngokomlinganiselo zingaphi izihlandlo owakhe wabelana ngesondo ngenyanga ngasemva?

☐ Izihlandlo

80) <u>Kwiinyanga ezi-6 ezidlulileyo</u>, ngoku <u>nanisabelana ngesondo ngemva, mangaphi amaxesha umlingane wakhe wayesebenzisa ngawo ikhondom</u>? (Nceda ukhethe ibhokisi enye kuphela)

☐ Soloko

☐ Ngaphezu kwesiqingatha sexesha

☐ Isiqingatha sexesha

☐ Ngaphantsi kwesiqingatha sexesha

☐ Zange

81) Ingaba iqabane lakho nakhe nasebenzisa iikhondom ukugqibela kwenu ukwabelana ngesondo ngemva? (Nceda uphawule ibhokisi ibenye kuphela)

☐ Ewe

☐ Hayi

82) Wakhe wafumana izipho, imali okanye iziyobisi ezisuka emadodeni kuba befuna ukulala nawe?

☐ Ewe

☐ Hayi (Ndiyabulela ngexesha lakho – nceda luphelise uphando)

83) <u>Kwiinyanga ezi-6 ezidlulileyo</u>, ingaba ikhona na indoda eyakhe yakupha <u>izipho, imali okanye iziyobisi kuba befuna ukulala nawe</u>?

☐ Ewe

☐ Hayi (Ndiyabulela ngexesha lakho – nceda luphelise uphando)

84) Kwiinyanga ezi-6 ezidlulileyo, mangaphi amaxesha amadoda ekupha izipho, imali okanye iziyobisi benaniselana ngoko kuba befuna ukulala nawe?

☐ Amaxesha

85) Kwiinyanga ezi-6 ezidlulileyo, ngoku ubunikwa izipho, imali, okanye iziyobisi zokuba wabelane nabo ngokulalana, ngomlomo, okanye xa kusabelwana ngesondo ngemva, nizisebenzise kangaphi wena okanye umlingane wakho iikhondom? (Nceda uphawule ibhokisi ibenye)

☐ Rhoqo

☐ Ngaphezu kwesiqingatha sexesha

☐ Isiqingatha sexesha

☐ Ngaphantsi kwesiqingatha sexesha

☐ Zange

SIYABULELA NGEXESHA LAKHO.
IGALELO LAKHO LIBALULEKE KAKHULU KWISIFUNDO SETHU.
UYASINCEDA UKUZE SIKWAZI UKUCWANGCISA NGCONO NGOKUKHATHALELWA KWEMPILO EKUHLALENI.
