# Supplementary material for: Incidence and risk factors of C. trachomatis and N. gonorrhoeae among young women from the Western Cape, South Africa: The EVRI study
Source: PLoS One. 2021 May 3;16(5):e0250871. doi: 10.1371/journal.pone.0250871 (PMC8092667; doi:10.1371/journal.pone.0250871)
Supplement: S4 File — (PDF) [file pone.0250871.s004.pdf]

Questions 1-4 to be filled in by the study coordinator:

1.Date of survey (day/month/year): |__|__| / |________________________/ |__|__|__|__|

2.PID #: | 7_|_0_|_ _|_ _|_ |

3.Date of birth (day/month/year): |__|__| /___________________________/ |__|__|__|__|

4.Study participant's initials: ___________

****

VISIT 4
STUDY QUESTIONNAIRE
THE EVRI STUDY

We appreciate your willingness to participate in this project.

All of the information you provide for us is strictly confidential, and your name will not be associated with this questionnaire and will never be used in reports.

Please provide the answer that best fits your situation.

5) What is your current marital status? (Please check only one box)

☐ Single, never married

☐ Married

☐ Living together

☐ Divorced/Separated

☐ Widowed

6) How many years of school have you completed? (Please check only one box)

☐ I never attended school

☐ I did not finish standard 5

☐ I am currently a grade 1-7 learner

☐ I left school before completing grade 7

☐ I am currently a grade 8-12 learner

☐ I left school and did not pass grade 12

☐ I have passed matric/grade 12 but did not attend college/technikon/university

☐ I did attend some college/technikon/university

☐ I am currently a student at college/technikon/university

☐ I have obtained a degree/diploma from college/technikon/university

7) Have you had at least one drink (beer, wine, or other drink with alcohol in it) in the past month?

☐ Yes

☐ No (Go to question 13)

8) During the past 1 month, on how many days did you have at least one drink with alcohol in it? (Please check only one box)

☐ One day

☐ Between 2-5 days

☐ More than 5 Days

9) On the days when you drank, about how many bottles of beer did you drink on average? (Enter 0 if none)

☐

10) On the days when you drank, about how many glasses of wine did you drink on average? (Enter 0 if none)

☐

11) On the days when you drank, about how many cocktails did you drink on average? (Enter 0 if none)

☐

12) On the days when you drank, about how many shots of liquor did you drink on average? (Enter 0 if none)

☐

13) Do you smoke cigarettes/roll-ups now?

- [ ] Yes
- [ ] No (Go to question 15)

14) How many cigarettes/roll-ups do you smoke per day?

- [ ] Number of cigarettes/roll-ups

15) Do you currently use chewing tobacco or snuff? (Please check only one box)

- [ ] Every Day
- [ ] Some Days
- [ ] Not at all

The following section will ask you questions about your reproductive health.

17) Are you currently using birth control (prevention)?

- [ ] Yes
- [ ] No (Go to question 20)

18) What type of birth control method are you using **now**? (*Check all that apply*)

- [ ] Oral contraceptive (“the pill”)
- [ ] Diaphragm
- [ ] IUD/The Loop/Coil
- [ ] Condoms
- [ ] Foam, cream, jelly, suppositories
- [ ] Depo Provera or other injectable contraceptives
- [ ] Rhythm method
- [ ] Withdrawal
- [ ] Sterilization (tubes tied)
- [ ] Vasectomy (male partner sterilized)
- [ ] Other

The next questions we are going to ask you are sensitive. Please note that some of the questions refer to the time BEFORE you started the study, and some of the questions refer to the time AFTER you started the study.

20) Since starting this study have you had a sex partner where you knew or thought that he had sexually transmitted disease (STD), or where you found out afterwards that he had a sexually transmitted disease (STD)?

- [ ] Yes
- [ ] No

21) Since starting this study have you had a sex partner who had HIV, or where you found out afterwards that he had HIV?

- [ ] Yes
- [ ] No

22) Since starting this study have you had a sex partner who had genital warts?

- [ ] Yes
- [ ] No
- [ ] Don’t know

23) Since starting this study have you had a male sex partner who was circumcised?

- [ ] Yes
- [ ] No
- [ ] Don’t know

24) Is your regular male sex partner circumcised? (Please check only one box)

- [ ] Yes
- [ ] No
- [ ] Don’t know
- [ ] Do not currently have a regular male sex partner

The following section will ask you questions about sexual relationships.

26) How old were you when you had vaginal sex for the first time?

☐ Years old

27) In your life, what is the number of men with whom you have had vaginal sex?

☐ Men

28) Since starting this study, how many different men have you had vaginal sex with?

☐ Men

29) Since starting this study, how many men have you had vaginal sex with for the first time?

☐ Men

30) Before starting this study, on average how often did you have vaginal sex? (Choose only one response)

☐ Less than once per month

☐ More than once a month

☐ More than once a week

31) Since starting this study, on average how often did you have vaginal sex? (Choose only one response)

☐ Less than once per month (Go to question 34)

☐ More than once a month (Go to question 33)

☐ More than once a week (Go to question 32)

32) Since starting this study, on average how many times per week did you have vaginal sex?

☐ Times per week (Go to question 34)

33) Since starting this study, on average how many times per month did you have vaginal sex?

☐ Times per month

34) Before starting this study, when you had vaginal sex, how often did you or your partner use condoms? (Please check only one box)

- [ ] Always
- [ ] More than half the time
- [ ] Half the time
- [ ] Less than half the time
- [ ] Never

35) Since starting this study, when you had vaginal sex, how often did you or your partner use condoms? (Please check only one box)

- [ ] Always
- [ ] More than half the time
- [ ] Half the time
- [ ] Less than half the time
- [ ] Never

36) Did you or your partner use condoms the last time you had vaginal sex? (Please check only one box)

- [ ] Yes
- [ ] No
- [ ] Don’t remember
- [ ] Never used a condom with vaginal sex

37) Before starting this study, did you performed oral sex on a man?

- [ ] Yes
- [ ] No

38) Since starting this study, did you perform oral sex on a man?

- [ ] Yes
- [ ] No (Go to question 42)

39) Since starting this study, on average how often did you perform oral sex on a man? (Choose only one response)

- ☐ Less than once a month (Go to question 42)
- ☐ More than once a month (Go to question 41)
- ☐ More than once a week (Go to question 40)

40) Since starting this study, on average how many times per week did you perform oral sex on a man?

- ☐ Times (Go to question 42)

41) Since starting this study, on average how many times per month did you perform oral sex on a man?

- ☐ Times

42) Before starting this study, did a man performed oral sex on you?

- ☐ Yes
- ☐ No

43) Since starting this study, did a man perform oral sex on you?

- ☐ Yes
- ☐ No (Go to question 47)

44) Since starting this study, how often did a man perform oral sex on you? (Choose only one response)

- ☐ Less than once a month (Go to question 47)
- ☐ More than once a month (Go to question 46)
- ☐ More than once a week (Go to question 45)

45) Since starting this study, how many times per week did a man perform oral sex on you?

- ☐ Times (Go to question 47)

46) Since starting this study, how many times per month did a man perform oral sex on you?

- ☐ Times

47) Before starting this study, did you have anal sex?

☐ Yes

☐ No

48) Since starting this study, did you have anal sex?

☐ Yes

☐ No (Go to question 54)

49) Since starting this study, on average how often did you have anal sex?
(Choose only one response

☐ Less than once a month (Go to question 52)

☐ More than once a month (Go to question 51)

☐ More than once a week (Go to question 50)

50) Since starting this study, on average how many times per week did you have anal sex?

☐ Times (Go to question 52)

51) Since starting this study, on average how many times per month did you have anal sex?

☐ Times

52) Since starting this study, when you had anal sex, how often did your partner use condoms?
(Please check only one box)

☐ Always

☐ More than half the time

☐ Half the time

☐ Less than half the time

☐ Never

53) Did your partner use condoms the last time you had anal sex? (Please check only one box)

☐ Yes

☐ No

☐ Don't remember

54) Since starting this study, has a man given you presents, money, or drugs in exchange for sex?

☐ Yes

☐ No (Go to question 58)

55) Since you started this study, how many times has a man given you presents, money, or drugs in exchange for sex with you?

☐ Times

56) Since you started this study, when you were given presents, money, or drugs for sex, how often did you or your partner use condoms? (Please check only one box)

☐ Always

☐ More than half the time

☐ Half the time

☐ Less than half the time

☐ Never

We are interested in your thoughts about the study so that we can improve. The next section asks questions about your participation in the study. Please answer as honestly as you can.

58) Before you agreed to take part in this study, was the purpose of the study clearly explained to you?

☐ Yes

☐ No

59) Was the money we gave you for travel enough to cover your expenses?

☐ Yes

☐ No

60) When you took the survey on the tablet computer did you find the tablet:

- [ ] Easy to use
- [ ] OK but needed a little help
- [ ] Difficult, I needed a lot of help
- [ ] I needed help but preferred not to get help

61) Would you have preferred a paper questionnaire to the tablet?

- [ ] Yes, prefer paper
- [ ] No, prefer the tablet

62) Were the questions on the survey easy to understand?

- [ ] Yes, all of them were easy to understand
- [ ] Yes, most of them were easy to understand
- [ ] No, many were difficult to understand

63) What is your home language?

- [ ] English
- [ ] Afrikaans
- [ ] Xhosa
- [ ] Other

64) Which language are you most comfortable speaking?

- [ ] English
- [ ] Afrikaans
- [ ] Xhosa
- [ ] Other

65) Which language are you most comfortable reading?

- [ ] English
- [ ] Afrikaans
- [ ] Xhosa
- [ ] Other

66) Did you complete the survey in Xhosa?

- [ ] Yes
- [ ] No (Go to question 68)

67) Was the Xhosa terminology:

- [ ] Easy to understand
- [ ] Difficult to understand
- [ ] Inappropriate terminology

68) Were you under the age of 18 at the start of this study?

- [ ] Yes
- [ ] No (Go to question 70)

69) Who accompanied you to the clinic and provided consent for you to participate?

- [ ] Mother
- [ ] Grandmother
- [ ] Other

70) When you signed the consent form, did you ask questions?

- [ ] No, I understood everything and did not have any questions
- [ ] No, I was afraid to ask questions
- [ ] Yes, I asked questions

71) What can we do to make the gynae exam more comfortable? (Check all that apply)

- [ ] It was fine as is
- [ ] Provide more privacy
- [ ] It would not matter, it will always be uncomfortable

72) Did you feel uncomfortable about being tested for HIV?

- [ ] Yes
- [ ] No

73) Have you learned more about sexually transmitted diseases from your participation in this study?

- [ ] Yes, a lot more
- [ ] Yes, somewhat more
- [ ] No

74) How have you changed your behaviours since participating in this study? (Check all that apply)

- [ ] I use condoms more often
- [ ] I use condoms every time I have sex
- [ ] I talk with my family or friends about STDs
- [ ] I talk with my sexual partner about STDs
- [ ] I have not changed my behaviours

75) Would you consider taking part in another study like this one in the future?

- [ ] Yes
- [ ] No

76) Do you wish to receive feedback as to the results of this study?

- [ ] Yes
- [ ] No

THANK YOU FOR YOUR TIME.
YOUR CONTRIBUTION IS VERY IMPORTANT TO OUR STUDY.
YOU ARE HELPING US TO PLAN FOR BETTER HEALTH CARE IN THE COMMUNITY.
