# Supplementary material for: Incidence and risk factors of C. trachomatis and N. gonorrhoeae among young women from the Western Cape, South Africa: The EVRI study
Source: PLoS One. 2021 May 3;16(5):e0250871. doi: 10.1371/journal.pone.0250871 (PMC8092667; doi:10.1371/journal.pone.0250871)
Supplement: S5 File — (PDF) [file pone.0250871.s005.pdf]

**Die studiekoördineerder moet vraag 1–4 invul.**

1. Datum waarop opname gedoen is (dag/maand/jaar): |__|__|/|____________/|__|__|__|__|

2. PID #: | 7_|_0_|_ _|_ _|_ |

3. Geboortedatum (dag/maand/jaar): |__|__|/________________________/|__|__|__|__|

4. Voorletters van deelnemer aan studie: ___________

***************************************************************************************************************

# BESOEK 4
# NAVORSINGSVRAELYS
# DIE EVRI-STUDIE

Ons waardeer dit dat jy bereid is om aan hierdie projek deel te neem.

Alle inligting wat jy aan ons verskaf, sal streng vertroulik bly. Jou naam sal nie met jou vraelys verbind word nie, en sal nooit in verslae genoem word nie.

**Gee asseblief by elke vraag die antwoord wat jou situasie die beste beskryf.**

5) Wat is jou huwelikstaat op die oomblik? (Merk asseblief net een blokkie.)

- ☐ Enkellopend, en was nog nooit getroud nie
- ☐ Getroud
- ☐ In ’n saamwoonverhouding
- ☐ Geskei of vervreemd
- ☐ Wewenaar/weduwee

6) Hoeveel jaar van skoolonderrig het jy voltooi? (Merk asseblief net een blokkie.)

- ☐ Ek het geen skoolopleiding nie.
- ☐ Ek het nie standerd 5 voltooi nie.
- ☐ Ek is op die oomblik ’n leerder in graad 1-7.
- ☐ Ek het uit die skool gegaan voordat ek graad 7 voltooi het.
- ☐ Ek is op die oomblik ’n leerder in graad 8-12.
- ☐ Ek het uit die skool gegaan sonder dat ek graad 12 voltooi het.

☐ Ek het matriek/graad 12 deurgekom, maar nie kollege/tegnikon/universiteit toe gegaan nie.

☐ Ek het 'n ruk aan 'n kollege/tegnikon/universiteit studeer.

☐ Ek studeer op die oomblik aan 'n kollege/tegnikon/universiteit.

☐ Ek het 'n graad/diploma aan 'n kollege/tegnikon/universiteit behaal.

7) Het jy in die afgelope maand minstens een drankie (bier, wyn of ander drank wat alkohol bevat) gehad?

☐ Ja

☐ Nee (Gaan na vraag 13.)

8) Hoeveel dae in **die afgelope maand** het jy minstens een drankie gehad wat alkohol bevat? (Merk asseblief net een blokkie.)

☐ Een dag

☐ Tussen 2 en 5 dae

☐ Meer as 5 dae

9) Ongeveer hoeveel bottels bier het jy gemiddeld gedrink op die dae wanneer jy iets gedrink het? (Indien geen, skryf 0.)

☐

10) Ongeveer hoeveel glase wyn het jy gemiddeld gedrink op die dae wanneer jy iets gedrink het? (Indien geen, skryf 0.)

☐

11) Ongeveer hoeveel mengeldrankies het jy gemiddeld gedrink op die dae wanneer jy iets gedrink het? (Indien geen, skryf 0.)

☐

12) Ongeveer hoeveel sopies sterk drank het jy gemiddeld gedrink op die dae wanneer jy iets gedrink het? (Indien geen, skryf 0.)

☐

13) Rook jy op die oomblik sigarette/selfgerolde sigarette?

- [ ] Ja
- [ ] Nee (Gaan na vraag 15.)

14) Hoeveel sigarette/selfgerolde sigarette rook jy per dag?

- [ ] sigarette/selfgerolde sigarette

15) Pruim of snuif jy op die oomblik tabak? (Merk asseblief net een blokkie.)

- [ ] Elke dag
- [ ] Party dae
- [ ] Glad nie

**Die volgende afdeling bevat vrae oor jou seksuele gesondheid.**

17) Gebruik jy op die oomblik ’n voorbehoedmiddel?

- [ ] Ja
- [ ] Nee (Gaan na vraag 20.)

18) Watter soort voorbehoedmiddel gebruik jy **op die oomblik**? (Merk alle blokkies wat van toepassing is.)

- [ ] Mondelikse voorbehoedmiddel (‘die pil’)
- [ ] Diafragma
- [ ] IUA/‘lussie’/‘veertjie’
- [ ] Kondome
- [ ] Skuim, room, jel, setpille
- [ ] Depo Provera of ander voorbehoedmiddels wat ingespuit word
- [ ] Ritmemetode
- [ ] Onttrekking voor saadstorting
- [ ] Sterilisasie (buise afgebind)
- [ ] Vasektomie (manlike maat gesteriliseer)
- [ ] Ander

**Die vrae wat nou volg, gaan oor sensitiewe sake. Let asseblief daarop dat party van die vrae handel oor die tydperk VOORDAT jy aan hierdie projek deelgeneem het, en party vrae handel oor die tydperk VANDAT jy hieraan deelneem.**

20) Het jy **vandat jy aan hierdie projek deelneem** seks gehad met iemand van wie jy geweet of vermoed het dat die persoon ’n seksueel oordraagbare siekte (SOS) het, of van wie jy agterna uitgevind het dat die persoon ’n SOS het?

☐ Ja

☐ Nee

21) Het jy **vandat jy aan hierdie projek deelneem** seks gehad met iemand wat MIV het, of van wie jy agterna uitgevind het dat die persoon MIV het?

☐ Ja

☐ Nee

22) Het jy **vandat jy aan hierdie projek deelneem** seks gehad met iemand wat vratte op sy of haar geslagsdele het?

☐ Ja

☐ Nee

☐ Weet nie

23) Het jy **vandat jy aan hierdie projek deelneem** seks gehad met ’n man wat besny is?

☐ Ja

☐ Nee

☐ Weet nie

24) Is die man met wie jy gereeld seks het, besny? (Merk asseblief net een blokkie.)

☐ Ja

☐ Nee

☐ Weet nie

☐ Ek het op die oomblik met geen man gereeld seks nie

**Die volgende afdeling bevat vrae oor jou seksuele verhoudings.**

26) Hoe oud was jy toe jy die eerste keer **vaginale seks** gehad het?

☐ jaar

27) Met hoeveel mans het jy al altesaam in jou lewe **vaginale seks** gehad?

☐ mans

28) Met hoeveel verskillende mans het jy **vandat jy aan hierdie projek deelneem vaginale seks** gehad?

☐ mans

29) Met hoeveel mans het jy **vandat jy aan hierdie projek deelneem** vir die eerste keer **vaginale seks** gehad?

☐ mans

30) Ongeveer hoe dikwels het jy **vaginale seks** gehad **voordat jy aan hierdie projek deelgeneem het**? (Kies net een opsie.)

☐ Minder as een keer per maand

☐ Meer as een keer per maand

☐ Meer as een keer per week

31) Ongeveer hoe dikwels het jy **vaginale seks** gehad **vandat jy aan hierdie projek deelneem**? (Kies net een opsie.)

☐ Minder as een keer 'n maand (Gaan na vraag 34.)

☐ Meer as een keer 'n maand (Gaan na vraag 33.)

☐ Meer as een keer 'n week (Gaan na vraag 32.)

32) Ongeveer hoeveel keer 'n week het jy **vaginale seks vandat jy aan hierdie studie deelneem**?

☐ keer per week (Gaan na vraag 34.)

33) Ongeveer hoeveel keer 'n maand het jy **vaginale seks vandat jy aan hierdie studie deelneem**?

☐ keer 'n maand

34) Hoe gereeld het julle ’n kondoom gebruik wanneer jy en jou maat **vaginale seks** gehad het **voordat jy aan hierdie projek deelgeneem het**? (Merk asseblief net een blokkie.)

- ☐ Altyd
- ☐ Meer as die helfte van die kere
- ☐ Die helfte van die kere
- ☐ Minder as die helfte van die kere
- ☐ Nooit

35) Hoe gereeld het julle ’n kondoom gebruik wanneer jy en jou maat **vaginale seks** gehad het **vandat jy aan hierdie projek deelneem**? (Merk asseblief net een blokkie.)

- ☐ Altyd
- ☐ Meer as die helfte van die kere
- ☐ Die helfte van die kere
- ☐ Minder as die helfte van die kere
- ☐ Nooit

36) Het julle ’n kondoom gebruik die vorige keer wat jy en jou maat **vaginale seks** gehad het? (Merk asseblief net een blokkie.)

- ☐ Ja
- ☐ Nee
- ☐ Kan nie onthou nie
- ☐ Het nog nooit ’n kondoom tydens **vaginale seks** gebruik nie

37) Het jy al ooit **voordat jy aan hierdie projek deelgeneem het**, vir ’n man **orale seks** gegee?

- ☐ Ja
- ☐ Nee

38) Het jy **vandat jy aan hierdie projek deelneem** vir ’n man **orale seks** gegee?

- ☐ Ja
- ☐ Nee (Gaan na vraag 42.)

39) Ongeveer hoe dikwels het jy **vandat jy aan hierdie projek deelneem** vir ’n man **orale seks** gegee? (Kies net een opsie.)

☐ Minder as een keer ’n maand (Gaan na vraag 42.)

☐ Meer as een keer ’n maand (Gaan na vraag 41.)

☐ Meer as een keer ’n week (Gaan na vraag 40.)

40) Ongeveer hoeveel keer ’n week gee jy **vandat jy aan hierdie projek deelneem** vir ’n man **orale seks**?

☐ keer (Gaan na vraag 42.)

41) Ongeveer hoeveel keer ’n maand gee jy **vandat jy aan hierdie projek deelneem** vir ’n man **orale seks**?

☐ keer

42) Het ’n man al ooit **voordat jy aan hierdie projek deelgeneem het vir jóú orale seks** gegee?

☐ Ja

☐ Nee

43) Het ’n man vir jou **orale seks** gegee **vandat jy aan hierdie projek deelneem**?

☐ Ja

☐ Nee (Gaan na vraag 47.)

44) Hoe dikwels gee ’n man vir jou **orale seks vandat jy aan hierdie projek deelneem**? (Kies net een opsie.)

☐ Minder as een keer ’n maand (Gaan na vraag 47.)

☐ Meer as een keer ’n maand (Gaan na vraag 46.)

☐ Meer as een keer ’n week (Gaan na vraag 45.)

45) Hoeveel keer ’n week gee ’n man vir jou **orale seks vandat jy aan hierdie projek deelneem**?

☐ keer (Gaan na vraag 47.)

46) Hoeveel keer ’n maand gee ’n man vir jou **orale seks vandat jy aan hierdie projek deelneem**?

☐ keer

47) Het jy ooit **voordat jy aan hierdie projek deelgeneem het anale seks** gehad?

- [ ] Ja
- [ ] Nee

48) Het jy **vandat jy aan hierdie projek deelneem anale seks** gehad?

- [ ] Ja
- [ ] Nee (Gaan na vraag 54.)

49) Ongeveer hoe dikwels het jy **anale seks vandat jy aan hierdie projek deelneem**? (Kies net een opsie.)

- [ ] Minder as een keer 'n maand (Gaan na vraag 52.)
- [ ] Meer as een keer 'n maand (Gaan na vraag 51.)
- [ ] Meer as een keer 'n week (Gaan na vraag 50.)

50) Ongeveer hoeveel keer 'n week het jy **anale seks vandat jy aan hierdie studie deelneem**?

- [ ] keer (Gaan na vraag 52.)

51) Ongeveer hoeveel keer 'n maand het jy **anale seks vandat jy aan hierdie studie deelneem**?

- [ ] keer

52) Hoe dikwels het julle 'n kondoom gebruik wanneer jy en jou maat **anale seks** gehad het **vandat jy aan hierdie projek deelneem**? (Merk asseblief net een blokkie.)

- [ ] Altyd
- [ ] Meer as die helfte van die kere
- [ ] Die helfte van die kere
- [ ] Minder as die helfte van die kere
- [ ] Nooit

53) Het jou maat ’n kondoom gebruik die vorige keer wat julle **anale seks** gehad het? (Merk asseblief net een blokkie.)

- [ ] Ja
- [ ] Nee
- [ ] Kan nie onthou nie

54) **Het ’n man jou vandat jy aan hierdie projek deelneem geskenke, geld of dwelmmiddels in ruil vir seks gegee?**

- [ ] Ja
- [ ] Nee (Gaan na vraag 58.)

55) **Hoeveel keer het ’n man jou vandat jy aan hierdie projek deelneem geskenke, geld of dwelmmiddels gegee in ruil daarvoor om seks met jou te hê?**

- [ ] keer

56) Hoe gereeld het julle ’n kondoom gebruik, **vandat jy aan hierdie projek deelneem**, wanneer jy geskenke, geld of dwelmmiddels in ruil vir seks gekry het? (Merk asseblief net een blokkie.)

- [ ] Altyd
- [ ] Meer as die helfte van die kere
- [ ] Die helfte van die kere
- [ ] Minder as die helfte van die kere
- [ ] Nooit

**Ons wil graag hoor wat jy dink, sodat ons kan verbeter. Die volgende afdeling vrae gaan oor jou deelname aan hierdie navorsingsprojek. Antwoord asseblief so eerlik as wat jy kan.**

58) Voordat jy ingestem het om aan hierdie projek deel te neem, het iemand die doel van die navorsing mooi vir jou verduidelik?

- [ ] Ja
- [ ] Nee

59) Was die geld wat ons vir jou vir vervoerkoste gegee het, genoeg om daarvoor te betaal?

- [ ] Ja
- [ ] Nee

60) Toe jy die opname op die tabletrekenaar gedoen het, hoe was dit vir jou om met die tablet te werk?

- [ ] Maklik om te gebruik
- [ ] Redelik maklik, maar 'n bietjie hulp was nodig
- [ ] Moeilik; ek het baie hulp nodig gehad
- [ ] Ek het hulp nodig gehad, maar het liewer nie daarvoor gevra nie

61) Sou jy verkies het om die vraelys op papier in te vul eerder as met die tablet?

- [ ] Ja, ek verkies papier
- [ ] Nee, ek verkies die tablet

62) Was die vrae in die opname maklik verstaanbaar?

- [ ] Ja, ek het almal maklik verstaan
- [ ] Ja, ek kon die meeste maklik verstaan
- [ ] Nee, baie was vir my moeilik om te verstaan

63) Wat is jou huistaal?

- [ ] Engels
- [ ] Afrikaans
- [ ] Xhosa
- [ ] Ander

64) Met watter taal voel jy die gemaklikste as jy moet praat?

- [ ] Engels
- [ ] Afrikaans
- [ ] Xhosa
- [ ] Ander

65) Met watter taal voel jy die gemaklikste as jy lees?

- ☐ Engels
- ☐ Afrikaans
- ☐ Xhosa
- ☐ Ander

66) Het jy hierdie opname in Xhosa gedoen?

- ☐ Ja
- ☐ Nee (Gaan na vraag 68.)

67) Hoe was die Xhosa-woorde wat oor gesondheidswetenskap gaan?

- ☐ Maklik om te verstaan
- ☐ Moeilik om te verstaan
- ☐ Die wetenskaplike woorde het nie gewerk nie

68) Was jy jonger as 18 toe jy by hierdie projek betrokke geraak het?

- ☐ Ja
- ☐ Nee (Gaan na vraag 70.)

69) Wie het saam met jou kliniek toe gekom en toestemming gegee dat jy mag deelneem?

- ☐ Ma
- ☐ Ouma
- ☐ Ander

70) Het jy vrae gevra voordat jy die toestemmingsvorm onderteken het?

- ☐ Nee, ek het alles verstaan en het dus nie vrae gehad nie
- ☐ Nee, ek was bang om vrae te vra
- ☐ Ja, ek het vrae gevra

71) Wat kan ons doen wat die intieme ondersoek minder ongemaklik sal maak? (Merk alle blokkies wat van toepassing is.)

- ☐ Dit was goed net so
- ☐ Gee meer privaatheid
- ☐ Niks sal help nie; dit sal maar altyd ongemaklik bly

72) Het dit jou ongemaklik gemaak om vir MIV getoets te word?

- ☐ Ja
- ☐ Nee

73) Het jy meer oor seksueel oordraagbare siektes (SOS'e) geleer deurdat jy aan hierdie projek deelgeneem het?

- ☐ Ja, baie meer
- ☐ Ja, ietsie meer
- ☐ Nee

74) Hoe het jy jou gedrag verander vandat jy aan hierdie projek deelneem? (Merk alle blokkies wat van toepassing is.)

- ☐ Ek gebruik meer dikwels kondome.
- ☐ Ek gebruik kondome elke keer wanneer ek seks het.
- ☐ Ek praat met my familie en vriende oor SOS'e.
- ☐ Ek praat met my seksmaat oor SOS'e.
- ☐ My gedrag het nie verander nie.

75) Sal jy dit oorweeg om deel te neem as ons weer so 'n projek in die toekoms sou doen?

- ☐ Ja
- ☐ Nee

76) Wil jy oor die uitslae van hierdie navorsing ingelig word?

- ☐ Ja
- ☐ Nee

**DANKIE VIR JOU TYD.**
**JOU BYDRAE IS BAIE BELANGRIK VIR ONS NAVORSINGSPROJEK.**
**JY HELP ONS OM VIR BETER GESONDHEIDSORG IN DIE GEMEENSKAP TE BEPLAN.**
