# Supplementary material for: Incidence and risk factors of C. trachomatis and N. gonorrhoeae among young women from the Western Cape, South Africa: The EVRI study
Source: PLoS One. 2021 May 3;16(5):e0250871. doi: 10.1371/journal.pone.0250871 (PMC8092667; doi:10.1371/journal.pone.0250871)
Supplement: S6 File — (PDF) [file pone.0250871.s006.pdf]

<u>Imibuzo yoku-1-4 kufuneka izaliswe ngumququzeleli wesi sifundo:</u>

1. Umhla wodliwano-ndlebe (umhla/inyanga/unyaka): |__|__| / ________ _______________/ |__|__|__|__|

2. I-PID #: | 7_|_0_|_ _|_ _|_ |

3. Umhla wokuzalwa (umhla/inyanga/unyaka): |__|__| / __________________________ / |__|__|__|__|

4. Oonobumba bokuqala begama lalowo uthatha inxaxheba kwisifundo: __________

****

UTYELELO LWESI-4
IPHEPHA ELINEMIBUZO YESIFUNDO
ISIFUNDO SE-EVRI

Siyakuvuyela ukuba nomdla kwakho ukuthatha inxaxheba kule projekthi.

Zonke iinkcukacha osinika zona ziza kuba yimfihlelo, kwaye igama lakho alizi kunxulunyaniswa neli phepha lemibuzo kwaye soze lisetyenziswe kwiingxelo.

Nceda usinike impendulo elungelelana nemeko yakho.

5) Sithini isimo sakho somtshato? (Nceda uphawule ibhokisi ibenye)

- ☐ Awutshatanga, zange utshate
- ☐ Utshatile
- ☐ Nihlala kunye
- ☐ Niqhawule umtshato/nahlukene
- ☐ Ngumhlolokazi

6) Uphele kubani esikolweni? (Nceda uphawule ibhokisi ibenye)

- ☐ Zange ndiye esikolweni
- ☐ Khange ndiligqibe ibanga lesi-5
- ☐

- [ ] Ngoku ndingumfundi okwibakala loku-1-7
- [ ] Ndisishiye isikolo phambi kokuba ndigqibe ibakala lesi-7
- [ ] Ngoku ndingumfundi okwibakala lesi-8-12
- [ ] Ndisiyekile isikolo kwaye khange ndiliphumelele ibakala le-12
- [ ] Ndilipasile ibanga leshumi/ibakala le-12 kodwa khange ndiye ekholejini/eteknikhoni/eyunivesithi
- [ ] Ndiyile ngaphambili ekholejini/eteknikhoni/eyunivesithi
- [ ] Ngoku ndingumfundi wasekholejini/eteknikhoni/eyunivesithi
- [ ] Ndifumene isidanga/idiploma ekholejini/eteknikhoni/eyunivesithi

7) Ubukhe wasela ubuncinane isiselo esinye (sebhiya, sewayini, okanye esinye isiselo esinxilisayo) kwinyanga enye edlulileyo?

- [ ] Ewe
- [ ] Hayi (Yiya kumbuzo we-13)

8) Kwinyanga e-1 edlulileyo, zingaphi iintsuku obukhe wasela ngazo ubuncinane isiselo esinye esinxilisayo? (Nceda uphawule ibhokisi ibenye)

- [ ] Usuku olunye
- [ ] Phakathi kweentsuku ezi-2-5
- [ ] Ngaphezu kweentsuku ezi-5

9) Kwiintsuku osela ngazo, ubusela malunga nomlinganiselo weebhotile ezingaphi zebhiya? (Faka u-0 ukuba akukho nanye)

[ ]

10) Kwiintsuku osela ngazo, ubusela malunga nomlinganiselo weeglasi zewayini ezingaphi? (Faka u-0 ukuba akukho nanye)

[ ]

11) Kwiintsuku osela ngazo, ubusela malunga nomlinganiselo wemixube yewayini engaphi? (Faka u-0 ukuba akukho nanye)

[ ]

12) Kwiintsuku osela ngazo, ubusela malunga nomlinganiselo weethothi ezingaphi zotywala? (Faka u-0 ukuba akukho nanye)

13) Uyayitshaya na ngoku isigarethi/imirolo?

- [ ] Ewe
- [ ] Hayi (Yiya kumbuzo we-15)

14) Zingaphi iisigarethi/imirolo oyitshayayo ngosuku?

- [ ] Inani leesigarethi/imirolo

15) Ingaba ngoku usebenzisa icuba elihlafunwayo okanye isinifu? (Nceda uphawule ibhokisi enye kuphela)

- [ ] Yonke imihla
- [ ] Ngezinye iintsuku
- [ ] Zange

Eli candelo lilandelayo liza kukubuza imibuzo emalunga nempilo kwiimeko zakho zokuzala

17) Ingaba ngoku usebenzisa izinto zokucwangcisa inzala (ukuthintela inzala)?

- [ ] Ewe
- [ ] Hayi (Yiya kumbuzo wama-20)

18) Loluphi uhlobo lokuthintela ukuzala olusebenzisayo **ngoku**? (*Jonga konke okusebenzayo kuwe*)

- [ ] Izinto ezityiwayo zokucwangcisa ("iipilisi")
- [ ] Isivalo
- [ ] I-IUD/iluphu/Isixhobo esifakwa esibelekweni sokuthintela ukuzala
- [ ] Iikhondom
- [ ] Ugwebu, ikhrimu, ijeli, iyeza elifakwa ebufazini elinyibilikayo
- [ ] I-Depo okanye ezinye izinto zokucwangcisa ezifana neenaliti
- [ ] Indlela yokuzibalela ukuba uya nini na exesheni
- [ ] Ukuyikhupha
- [ ] Ukuvala (ukubotshwa kweetyhubhu)
- [ ] Ukuvalwa kwenzala ebudodeni (ukuvala komlingane wakhe inzala umphelo)
- [ ]

Olunye

Imibuzo elandelayo esiza kukubuza yona ivakala buthathaka. Nceda uqaphele ukuba eminye imibuzo ibhekisele kwixesha PHAMBI kokuba uqale kwesi sifundo, eminye imibuzo ibhekisele kwixesha EMVA kokuba uqale kwesi sifundo.

20) Ukususela oko kuqalwe esi sifundo wakhe wanalo iqabane owabelana nalo ngesondo apho wawusazi okanye ulicingela ukuba linesifo esosulela ngokwabelana ngesondo (iSTD), okanye apho uye wafumanisa mva ukuba ebenesifo esosulela ngokwabelana ngesondo (iSTD)?

☐ Ewe

☐ Hayi

21) Ukususela oko kuqalwe esi sifundo, wakhe wanalo iqabane owabelana ngesondo elakhe laneNtsholongwane kaGawulayo, okanye apho wafumanisa kamva ukuba lineNtsholongwane kaGawulayo?

☐ Ewe

☐ Hayi

22) Ukususela oko kuqalwe esi sifundo wakhe wanalo iqabane owabelana nalo ngesondo elakhe lanesifo esosulela ngesondo?

☐ Ewe

☐ Hayi

☐ Andazi

23) Ukususela oko kuqalwe esi sifundo wakhe wanalo iqabane eliyindoda owabelana nalo ngesondo elaye loluka?

☐ Ewe

☐ Hayi

☐ Andazi

24) Ingaba iqabane lakho eliyindoda owabelana nalo ngesondo rhoqo lolukile? (Nceda ukhethe ibhokisi enye kuphela)

☐ Ewe

☐ Hayi

☐ Andazi

☐ Andinalo ngoku iqabane eliyindoda endabelana nalo rhoqo ngesondo

Eli candela lilandelayo liza kukubuza imibuzo ngezinxulumene nezesondo,

26) Wawunangaphi ukuqala kwakho ukwabelana ngesondo ngokulalana?

☐ Iminyaka ubudala

27) Ebomini bakho, mangaphi amadoda okwakhe wabelana nawo ngesondo ngokulalana?

☐ Amadoda

28) Ukususela oko kuqalwe esi sifundo, mangaphi amadoda ohluka-hlukeneyo owakhe wabelana ngesondo ngokulalana nawo?

☐ Amadoda

29) Ukususela oko kuqalwe esi sifundo, mangaphi amadoda owakhe wabelana ngesondo ngokulalana nawo okokuqala?

☐ Amadoda

30) Phambi kokuba kuqalwe esi sifundo, ngokomlinganiselo mangaphi amadoda owakhe wabelana ngesondo ngokulalana nawo? (Nceda ukhethe impendulo ibenye kuphela)

☐ Ngaphantsi kwakanye ngenyanga

☐ Ngaphezu kwakanye ngenyanga

☐ Ngaphezu kwakanye ngeveki

31) Ukususela oko kuqalwe esi sifundo, ngokomlinganiselo ubusabelana kangaphi ngesondo ngokulalana? (Khetha impendulo ibenye kuphela)

☐ Ngaphantsi kwakanye ngenyanga (Yiya kumbuzo wama-34)

☐ Ngaphezu kwakanye ngenyanga (Yiya kumbuzo wama-33)

☐ Ngaphezu kwakanye ngeveki (Yiya kumbuzo wama-32)

32) Ukususela oko kuqalwe esi sifundo, ngokomlinganiselo zingaphi izihlandlo ngeveki okhe wabelana ngesondo ngokulalana?

☐ Izihlandlo ngeveki (Yiya kumbuzo wama-34)

33) Ukususela oko kuqalwe esi sifundo, ngokomlinganiselo zingaphi izihlandlo ngenyanga okhe wabelana ngesondo ngokulalana?

☐ Izihlandlo ngenyanga

34) Phambi kokuqalwa kwesi sifundo, ngoku wawusabelana ngesondo ngokulalana, wena okanye iqabane lakho naniyisebenzisa kangaphi ikhondom? (Nceda uphawule ibhokisi ibenye kuphela)

☐ Soloko
☐ Ngaphezu kwesiqingatha sexesha
☐ Isiqingatha sexesha
☐ Ngaphantsi kwesiqingatha sexesha
☐ Zange

35) Ukususela oko kuqalwe, esi sifundo, ngoku wawusabelana ngesondo ngokulalana, wena okanye iqabane lakho nanizisebenzisa kangaphi iikhondom? (Nceda uphawule ibhokisi ibenye kuphela)

☐ Soloko
☐ Ngaphezu kwesiqingatha sexesha
☐ Isiqingatha sexesha
☐ Ngaphantsi kwesiqingatha sexesha
☐ Zange

36) Ingaba wena okanye iqabane lakho nakhe nasebenzisa iikhondom kwixesha elidlulileyo nisabelana ngesondo ngokulalana? (Nceda uphawule ibhokisi ibenye kuphela)

☐ Ewe
☐ Hayi

☐ Andisakhumbuli

☐ Zange ndisebenzise khondom xa ndisabelana ngesondo ngokulalana

37) Phambi kokuqalwa kwesi sifundo, wakhe wabelana ngesondo ngomlomo nendoda?

☐ Ewe

☐ Hayi

38) Ukususela oko kuqalwe esi sifundo, wakhe wabelana ngesondo ngomlomo nendoda?

☐ Ewe

☐ Hayi (Yiya kumbuzo wama-42)

39) Ukususela oko kuqalwe esi sifundo, ngokomlinganiselo wakhe wabelana ngesondo kangaphi ngomlomo nendoda? (Khetha impendulo ibenye kuphela)

☐ Ngaphantsi kwakanye ngenyanga (Yiya kumbuzo wama-42)

☐ Ngaphezu kwakanye ngenyanga (Yiya kumbuzo wama-41)

☐ Ngaphezu kwakanye ngeveki (Yiya kumbuzo wama-40)

40) Ukususela oko kuqalwe esi sifundo, ngokomlinganiselo zingaphi izihlandlo owakhe wabelana ngesondo ngeveki ngomlomo nendoda?

☐ Izihlandlo (Yiya kumbuzo wama-42)

41) Ukususela oko kuqalwe esi sifundo, ngokomlinganiselo zingaphi izihlandlo owakhe wabelana ngesondo ngenyanga ngomlomo nendoda?

☐ Izihlandlo

42) Phambi kokuqalwa kwesi sifundo, ingaba yakhe indoda yabelana ngesondo ngomlomo nawe?

☐ Ewe

☐ Hayi

43) Ukususela oko kuqalwe esi sifundo, ingaba yakhe indoda yabelana ngesondo ngomlomo nawe?

☐ Ewe

☐ Hayi (Yiya kumbuzo wama-47)

44) Ukususela oko kuqalwe esi sifundo, mangaphi amaxesha indoda isabelana ngesondo ngomlomo nawe? (Khetha impendulo ibenye kuphela)

☐ Ngaphantsi kwakanye ngenyanga (Yiya kumbuzo wama-47)

☐ Ngaphezu kwakanye ngenyanga (Yiya kumbuzo wama-46)

☐ Ngaphezu kwakanye ngeveki (Yiya kumbuzo wama-45)

45) Ukususela oko kuqalwe esi sifundo, zingaphi izihlandlo owakhe wabelana ngesondo ngeveki ngomlomo?

☐ Izihlandlo (Yiya kumbuzo wama-47)

46) Ukususela oko kuqalwe esi sifundo, zingaphi izihlandlo owakhe wabelana ngesondo ngenyanga nendoda?

☐ Amaxesha

47) Phambi kokuqalwa kwesi sifundo, wakhe wabelana ngesondo ngemva?

☐ Ewe

☐ Hayi

48) Ukususela oko kuqalwe esi sifundo, wakhe wabelana ngesondo ngemva?

☐ Ewe

☐ Hayi (Yiya kumbuzo wama-54)

49) Ukususela oko kuqalwe esi sifundo, ngokomlinganiselo kukangaphi usabelana ngesondo ngemva?
(Khetha impendulo ibenye kuphela)

☐ Ngaphantsi kwakanye ngenyanga (Yiya kumbuzo wama-52)

☐ Ngaphezu kwakanye ngenyanga (Yiya kumbuzo wama-51)

☐ Ngaphezu kwakanye ngeveki (Yiya kumbuzo wama-50)

50) Ukususela oko kuqalwe esi sifundo, ngokomlinganiselo zingaphi izihlandlo ngeveki usabelana ngesondo ngemva?

☐ Amaxesha (Yiya kumbuzo wama-52)

51) Ukususela oko kuqalwe esi sifundo, ngokomlinganiselo zingaphi izihlandlo ngenyanga usabelana ngesondo ngemva?

☐ Amaxesha

52) Ukususela oko kuqalwe esi sifundo, ngoku nanisabelana ngesondo ngemva, mangaphi amaxesha umlingane wakho wayesebenzisa ngawo iikhondom? (Nceda ukhethe ibhokisi enye kuphela)

☐ Soloko

☐ Ngaphezu kwesiqingatha sexesha

☐ Isiqingatha sexesha

☐ Ngaphantsi kwesiqingatha sexesha

☐ Zange

53) Ingaba iqabane lakho lakhe lasebenzisa iikhondom ukugqibela kwenu ukwabelana ngesondo ngemva? (Nceda uphawule ibhokisi ibenye kuphela)

☐ Ewe

☐ Hayi

☐ Andikhumbuli

54) Ukususela oko kuqalwe esi sifundo, ingaba ikhona na indoda eyakhe yakupha izipho, imali okanye iziyobisi kuba ifuna ukulala nawe?

☐ Ewe

☐ Hayi (Yiya kumbuzo wama-58)

55) Ukususela oko kuqalwe esi sifundo, mangaphi amaxesha amadoda ekupha izipho, imali okanye iziyobisi benaniselana ngoko kuba befuna ukulala nawe?

☐ Amaxesha

56) Ukususela oko kuqalwe esi sifundo, ngoku ubunikwa izipho, imali, okanye iziyobisi zokuba wabelane nabo ngokulalana, nizisebenzise kangaphi wena okanye umlingane wakho iikhondom? (Nceda uphawule ibhokisi ibenye)

- ☐ Rhoqo
- ☐ Ngaphezu kwesiqingatha sexesha
- ☐ Isiqingatha sexesha
- ☐ Ngaphantsi kwesiqingatha sexesha
- ☐ Zange

Sinomdla ngeengcinga zakho malunga nesi sifundo ukuze sisiphucule. Icandelo elilandelayo libuza imibuzo malunga nokuthatha kwakho inxaxheba kwesi sifundo. Nceda uphendule ngokunyanisekileyo kangangoko unako.

58) Phambi kokuba uvume ukuthatha inxaxheba kwesi sifundo, ingaba injongo yesifundo wayicaciselwa ngokucacileyo?

- ☐ Ewe
- ☐ Hayi

59) Ingaba imali esikunike yona ukuze ukwazi ukukhwela yayanele ukuzithwala ezo ndleko zakho?

- ☐ Ewe
- ☐ Hayi

60) Ukulufumana kwakho olu phando kwicwecwe lekhompyutha walifumana icwecwe:

- ☐ Kulula ukulisebenzisa
- ☐ Lilungile kodwa lidinga uncedo oluncinane
- ☐ Linzima, ndidinge uncedo olukhulu
- ☐ Ndidinge uncedo kodwa ndakhetha ukungalufuni olo ncedo

61) Ingaba ukhetha iphepha elinemibuzo kunecwecwe?

- ☐ Ewe, ndikhetha iphepha
- ☐ Hayi, ndikhetha icwecwe

62) Ingaba imibuzo yophando ilula ukuba ungayiqonda?

- [ ] Ewe, yonke bekulula ukuyiqonda
- [ ] Ewe, uninzi lwayo bekulula ukuyiqonda
- [ ] Hayi, uninzi lwayo bekunzima ukuyiqonda

63) Loluphi ulwimi lwakho lwasekhaya?

- [ ] IsiNgesi
- [ ] IsiBhulu
- [ ] IsiXhosa
- [ ] Olunye

64) Loluphi olona lwimi uziva ukhululekile ukuluthetha?

- [ ] IsiNgesi
- [ ] IsiBhulu
- [ ] IsiXhosa
- [ ] Olunye

65) Loluphi olona lwimi uziva ukhululekile ukulufunda?

- [ ] IsiNgesi
- [ ] IsiBhulu
- [ ] IsiXhosa
- [ ] Olunye

66) Ingaba ulwenze ngesiXhosa uphando?

- [ ] Ewe
- [ ] Hayi (Yiya kumbuzo wama-68)

67) Ingaba isigama sesiXhosa:

- [ ] Bekulula ukusiqonda
- [ ] Bekunzima ukusiqonda
- [ ] Ibisisigama esingafanelekanga

68) Ingaba ubungaphantsi kweminyaka eli-18 ukuqalwa kwesi sifundo?

- [ ] Ewe
- [ ] Hayi (Yiya kumbuzo wama-70)

69) Ukhatshwe ngubani ukuya ekliniki waze wakunika imvume yokuba uthathe inxaxheba?

- [ ] Umama
- [ ] Umakhulu
- [ ] Omnye

70) Xa ubutyikitya ifomu yesivumelwano, ikhona imibuzo oye wayibuza?

- [ ] Hayi, yonke into ndiyiqondile kwaye khange ndibe namibuzo
- [ ] Hayi, bendisoyika ukubuza imibuzo
- [ ] Ewe, ndibuze imibuzo

71) Singenza ntoni ukuzenza iimvavanyo zombelekisi zikwenze ukhululekile? (Jonga konke okusebenzayo)

- [ ] Bezilunge ngolu hlobo bezilulo
- [ ] Makubekho indawo yabucala
- [ ] Azinamsebenzi, ziza kuhlala zindenza ndingaziva ndikhululekile

72) Ingaba uzive ungakhululekanga xa ubuhlolwa ukuba awunaNtsholongwane kaGawulayo kusini na?

- [ ] Ewe
- [ ] Hayi

73) Ingaba ufunde ngakumbi malunga nezzifo ezosulela ngokwabelana ngesondo ngokuthatha kwakho inxaxheba kwesi sifundo?

☐ Ewe, kakhulu

☐ Ewe, pha napha

☐ Hayi

74) Uzitshintshe njani iindlela zokuziphatha ukususela oko uthatha inxaxheba kwesi sifundo? (Jonga konke okusebenzayo)

☐ Ndizisebenzisa rhoqo iikhondom

☐ Ndizisebenzisa ngalo lonke ixesha ndisabelana ngesondo iikhondom

☐ Ndiyathetha nosapho lwam okanye nezihlobo zam malunga nezifo ezosulela ngokwabelana ngesondo

☐ Ndiyathetha neqabane lam endabelana ngalo ngesondo malunga nezofo ezosulela ngokwabelana ngesondo

☐ Andizitshintshanga iindlela zam zokuziphatha

75) Ingaba ungakucinga ukuthatha inxaxheba kwesinye isifundo esifana nesi kwixesha elizayo?

☐ Ewe

☐ Hayi

76) Ingaba unqwenela ukuziva iziphumo zesi sifundo?

☐ Ewe

☐ Hayi

SIYABULELA NGEXESHA LAKHO.
IGALELO LAKHO LIBALULEKE KAKHULU KWISIFUNDO SETHU.
UYASINCEDA UKUZE SIKWAZI UKUCWANGCISA NGCONO NGOKUKHATHALELWA KWEMPILO EKUHLALENI.
