# Supplementary material for: Incidence and risk factors of C. trachomatis and N. gonorrhoeae among young women from the Western Cape, South Africa: The EVRI study
Source: PLoS One. 2021 May 3;16(5):e0250871. doi: 10.1371/journal.pone.0250871 (PMC8092667; doi:10.1371/journal.pone.0250871)
Supplement: S1 Table — (DOCX) [file pone.0250871.s007.docx]

**S1 Table. Sensitivity analysis of the determinants of an incident chlamydia, gonorrhoea, and chlamydia and/or gonorrhoea infection^a^, EVRI study, Cape Town, South Africa, October 2012-February 2014**

|  | **Incident chlamydia**  **(n=28)^b^** | | | | | | **Incident gonorrhoea**  **(n=29)^c^** | | | | | | **Incident chlamydia and/or gonorrhoea**  **(n=70)^d^** | | | | | | |
| --- | --- | --- | --- | --- | --- | --- | --- | --- | --- | --- | --- | --- | --- | --- | --- | --- | --- | --- | --- |
|  | IRR | (95% CI) | *p* | aIRR^e^ | (95% CI) | *p* | IRR | (95% CI) | *p* | aIRR^f^ | (95% CI) | *p* | | IRR | (95% CI) | *p* | aIRR^g^ | (95% CI) | *p* |
| ***1. Socio-demographic characteristics*** | | |  |  |  |  |  |  |  |  |  |  | |  |  |  |  |  |  |
| **Age, continuous^h^** | 0.47 | (0.08;2.84) | *0.41* | 0.61 | (0.06;5.70) | *0.66* | 1.17 | (0.18;7.37) | *0.87* | 1.31 | (0.15;11.82) | *0.81* | | 0.48 | (0.11;2.15) | *0.34* | 0.59 | (0.10;3.52) | *0.56* |
| **Age, categorical** |  |  |  |  |  |  |  |  |  |  |  |  | |  |  |  |  |  |  |
| < 20 year | REF |  | *0.53* |  |  |  | REF |  | *0.59* |  |  |  | | REF |  | *0.40* |  |  |  |
| 20 - 21 year | 0.89 | (0.37;2.11) |  |  |  |  | 1.44 | (0.60;3.48) |  |  |  |  | | 0.83 | (0.41;1.70) |  |  |  |  |
| ≥ 22 year | 0.60 | (0.24;1.52) |  |  |  |  | 0.92 | (0.34;2.47) |  |  |  |  | | 0.59 | (0.27;1.29) |  |  |  |  |
| **Race** |  |  |  |  |  |  |  |  |  |  |  |  | |  |  |  |  |  |  |
| Black | REF |  | *0.62* |  |  |  | REF |  | *0.72* |  |  |  | | REF |  | *0.73* |  |  |  |
| Other | 0.60 | (0.08;4.43) |  |  |  |  | 1.32 | (0.31;5.57) |  |  |  |  | | 1.24 | (0.38;4.05) |  |  |  |  |
| **Highest education level** | | |  |  |  |  |  |  |  |  |  |  | |  |  |  |  |  |  |
| ≤ Grade 12 | REF |  | *0.94* |  |  |  | REF |  | *0.09* |  |  |  | | REF |  | *0.65* |  |  |  |
| Passed grade 12 | 0.99 | (0.34;2.90) |  |  |  |  | 0.40 | (0.12;1.40) |  |  |  |  | | 0.74 | (0.31;1.75) |  |  |  |  |
| Some college/tech | 1.18 | (0.43;3.25) |  |  |  |  | 0.30 | (0.07;1.30) |  |  |  |  | | 0.70 | (0.28;1.75) |  |  |  |  |
| **Marital status** |  |  |  |  |  |  |  |  |  |  |  |  | |  |  |  |  |  |  |
| Single | REF |  | *0.72* |  |  |  | ^i^ |  |  |  |  |  | | REF |  | *0.46* |  |  |  |
| Other | 0.71 | (0.09;5.27) |  |  |  |  |  |  |  |  |  |  | | 0.51 | (0.07;3.74) |  |  |  |  |
| **Alcohol use in past month** | | |  |  |  |  |  |  |  |  |  |  | |  |  |  |  |  |  |
| No | REF |  | *0.31* |  |  |  | REF |  | *0.12* |  |  |  | | REF |  | *0.92* |  |  |  |
| Yes | 0.64 | (0.27;1.51) |  |  |  |  | 2.25 | (0.82;6.20) |  |  |  |  | | 1.04 | (0.51;2.10) |  |  |  |  |
| **Current smoker** |  |  |  |  |  |  |  |  |  |  |  |  | |  |  |  |  |  |  |
| No | REF |  | *0.26* |  |  |  | REF |  | *0.58* |  |  |  | | REF |  | *0.33* |  |  |  |
| Yes | 0.37 | (0.05;2.78) |  |  |  |  | 1.44 | (0.42;4.90) |  |  |  |  | | 0.52 | (0.12;2.19) |  |  |  |  |
| ***2. Sex related characteristics*** | | |  |  |  |  |  |  |  |  |  |  | |  |  |  |  |  |  |
| **Age at first sex act, continuous^h^** | 2.78 | (0.19;41.67) | *0.46* |  |  |  | 0.64 | (0.04;10.33) | *0.75* |  |  |  | | 1.12 | (0.12;10.88) | *0.92* |  |  |  |
| **Age at first sex act, categorical** | | |  |  |  |  |  |  |  |  |  |  | |  |  |  |  |  |  |
| < 16 year | REF |  | *0.49* | REF |  |  | REF |  | *0.43* | REF |  |  | | REF |  | *0.57* | REF |  |  |
| 16 - 17 year | 2.29 | (0.51;10.33) |  | 1.57 | (0.32;7.66) | *0.58* | 1.81 | (0.51;6.40) |  | 1.55 | (0.42;5.77) | *0.51* | | 1.75 | (0.59;5.16) |  | 1.42 | (0.46;4.39) | *0.54* |
| ≥ 18 year | 2.45 | (0.49;12.15) |  | 1.88 | (0.32;11.20) | *0.49* | 0.95 | (0.21;4.26) |  | 0.66 | (0.11;4.16) | *0.66* | | 1.30 | (0.38;4.45) |  | 1.28 | (0.32;5.07) | *0.73* |
| **Ever been pregnant** | | |  |  |  |  |  |  |  |  |  |  | |  |  |  |  |  |  |
| No | REF |  | *0.72* |  |  |  | REF |  | *0.36* |  |  |  | | REF |  | *0.28* |  |  |  |
| Yes | 1.16 | (0.52;2.58) |  |  |  |  | 1.44 | (0.66;3.14) |  |  |  |  | | 1.44 | (0.75;2.77) |  |  |  |  |
| **Current birth control use** | | |  |  |  |  |  |  |  |  |  |  | |  |  |  |  |  |  |
| No | REF |  | *0.27* |  |  |  | REF |  | *0.15* |  |  |  | | REF |  | *0.30* |  |  |  |
| Yes | 1.71 | (0.63;4.68) |  |  |  |  | 2.25 | (0.75;6.72) |  |  |  |  | | 1.51 | (0.68;3.36) |  |  |  |  |
| **Type of birth control** | | |  |  |  |  |  |  |  |  |  |  | |  |  |  |  |  |  |
| No current use | REF |  | *0.35* |  |  |  | REF |  | *0.08* |  |  |  | | REF |  | *0.45* |  |  |  |
| Condom | 2.22 | (0.78;6.31) |  |  |  |  | 1.63 | (0.48;5.58) |  |  |  |  | | 1.71 | (0.73;4.00) |  |  |  |  |
| Depo-Provera | 0.99 | (0.24;4.15) |  |  |  |  | 2.70 | (0.76;9.55) |  |  |  |  | | 0.98 | (0.32;3.01) |  |  |  |  |
| Oral contraceptives | 1.10 | (0.13;9.39) |  |  |  |  | 3.38 | (0.62;18.45) |  |  |  |  | | 0.55 | (0.55;7.81) |  |  |  |  |
| **Lifetime number of sex partners, continuous^j^** | 1.10 | (0.54;2.23) | *0.80* |  |  |  | 1.51 | (0.79;2.87) | *0.21* |  |  |  | | 1.37 | (0.81;2.35) | *0.24* |  |  |  |
| **Lifetime number of sex partners, categorical** |  |  |  |  |  |  |  |  |  |  |  |  | |  |  |  |  |  |  |
| < 3 | REF |  | *0.67* | REF |  |  | REF |  | *0.006* | REF |  |  | | REF |  | *0.19* | REF |  |  |
| 3 – 4 | 1.46 | (0.56;3.79) |  | 1.78 | (0.66;4.80) | *0.26* | 5.76 | (1.61;20.63) |  | 6.06 | (1.67;22.06) | *0.006* | | 1.98 | (0.87;4.52) |  | 2.28 | (0.97;5.39) | *0.06* |
| ≥ 5 | 0.92 | (0.28;3.00) |  | 0.79 | (0.21;2.98) | *0.73* | 4.65 | (1.16;18.59) |  | 4.32 | (1.06;17.55) | *0.041* | | 1.90 | (0.77;4.68) |  | 1.83 | (0.70;4.73) | *0.22* |
| **Sex partners since start study, continuous^j^** | 0.59 | (0.24;1.43) | *0.24* |  |  |  | 0.93 | (0.38;2.28) | *0.88* |  |  |  | | 0.60 | (0.28;1.26) | *0.17* |  |  |  |
| **Sex partners since start study, categorical** | | |  |  |  |  |  |  |  |  |  |  | |  |  |  |  |  |  |
| 0 | REF |  | *0.50* |  |  |  | REF |  | *0.92* |  |  |  | | REF |  | *0.23* |  |  |  |
| 1 | 1.31 | (0.37;4.59) |  |  |  |  | 1.31 | (0.29;5.92) |  |  |  |  | | 1.35 | (0.46;3.94) |  |  |  |  |
| ≥ 2 | 0.72 | (0.17;3.03) |  |  |  |  | 1.35 | (0.28;6.48) |  |  |  |  | | 0.67 | (0.20;2.28) |  |  |  |  |
| **Condom use since start study** | | |  |  |  |  |  |  |  |  |  |  | |  |  |  |  |  |  |
| No sex | REF |  | *0.77* |  |  |  | REF |  | *0.41* |  |  |  | | REF |  | *0.45* |  |  |  |
| Always | 0.85 | (0.23;3.15) |  |  |  |  | 1.15 | (0.25;5.30) |  |  |  |  | | 0.92 | (0.30;2.78) |  |  |  |  |
| More than half of the time | 1.41 | (0.29;7.00) |  |  |  |  | 1.10 | (0.15;7.81) |  |  |  |  | | 1.17 | (0.29;4.67) |  |  |  |  |
| Half of the time | 0.98 | (0.16;5.86) |  |  |  |  | 0.57 | (0.05;6.31) |  |  |  |  | | 0.61 | (0.11;3.34) |  |  |  |  |
| Less than half of the time or never | 1.88 | (0.42;8.42) |  |  |  |  | 2.77 | (0.56;13.73) |  |  |  |  | | 1.98 | (0.60;6.58) |  |  |  |  |
| **Sex for presents/money/drugs for sex since start study** | | |  |  |  |  |  |  |  |  |  |  | |  |  |  |  |  |  |
| No | ^i^ |  |  |  |  |  | REF |  | *0.29* |  |  |  | | REF |  | *0.78* |  |  |  |
| Yes |  |  |  |  |  |  | 2.21 | (0.51;9.54) |  |  |  |  | | 0.77 | (0.10;5.61) |  |  |  |  |

**Abbreviations:** aIRR, adjusted incidence ratio; CI, confidence interval; IRR, incidence rate ratio; REF, reference category

1. Excluding those participants with a chlamydia or gonorrhoea infection at enrolment
2. 8 incident events were excluded from multivariable analysis due to missing values
3. 7 incident events were excluded from multivariable analysis due to missing values
4. 10 incident events were excluded from multivariable analysis due to missing values
5. Variables included in the multivariable model based on univariable analysis: age, lifetime number of sex partners, age at first sex act
6. Variables included in the multivariable model based on univariable analysis: age, lifetime number of sex partners, age at first sex act
7. Variables included in the multivariable mode based on univariable analysis: age, lifetime number of sex partners, age at first sex act
8. Per 10 year increase in age
9. Excluded from univariable and multivariable analysis due to 0 observations in one of the categories
10. Per (log+1) increase in partner
