# Supplementary material for: Incidence and risk factors of C. trachomatis and N. gonorrhoeae among young women from the Western Cape, South Africa: The EVRI study
Source: PLoS One. 2021 May 3;16(5):e0250871. doi: 10.1371/journal.pone.0250871 (PMC8092667; doi:10.1371/journal.pone.0250871)
Supplement: Attachment — Submitted filename: Responses to reviewers.docx [file pone.0250871.s008.docx]

**[Note: HTML markup is below. Please do not edit.]

Reviewers' comments:

*Reviewer's Responses to Questions***

***Comments to the Author

1. Is the manuscript technically sound, and do the data support the conclusions?

The manuscript must describe a technically sound piece of scientific research with data that supports the conclusions. Experiments must have been conducted rigorously, with appropriate controls, replication, and sample sizes. The conclusions must be drawn appropriately based on the data presented.***

***Reviewer #1: Partly***

***Reviewer #2: Yes***

***2. Has the statistical analysis been performed appropriately and rigorously?***

***Reviewer #1: Yes***

***Reviewer #2: Yes***

***3. Have the authors made all data underlying the findings in their manuscript fully available?

The*** [***PLOS Data policy***](http://www.plosone.org/static/policies.action#sharing) ***requires authors to make all data underlying the findings described in their manuscript fully available without restriction, with rare exception (please refer to the Data Availability Statement in the manuscript PDF file). The data should be provided as part of the manuscript or its supporting information, or deposited to a public repository. For example, in addition to summary statistics, the data points behind means, medians and variance measures should be available. If there are restrictions on publicly sharing data—e.g. participant privacy or use of data from a third party—those must be specified.***

***Reviewer #1: Yes***

***Reviewer #2: Yes***

***4. Is the manuscript presented in an intelligible fashion and written in standard English?

PLOS ONE does not copyedit accepted manuscripts, so the language in submitted articles must be clear, correct, and unambiguous. Any typographical or grammatical errors should be corrected at revision, so please note any specific errors here.***

***Reviewer #1: Yes***

***Reviewer #2: Yes***

***5. Review Comments to the Author

Please use the space provided to explain your answers to the questions above. You may also include additional comments for the author, including concerns about dual publication, research ethics, or publication ethics. (Please upload your review as an attachment if it exceeds 20,000 characters)***

***Reviewer #1: The manuscript by Jongen et al. deals with the important topic of high prevalence and incidence rates of STIs among young women in South Africa. Syndromic management remains the main approach to STI care in South Africa, but studies like this highlight the urgent need to move to a diagnostic care approach. The authors describe a high burden of STIs in this cohort, and describe risk factors for incident infections. These results for the incidence analysis need to be interpreted with caution as no test of cure was conducted as part of the study to definitely exclude persistent infection. Overall the manuscript adds to the growing literature of STIs in South Africa, but I suggest that the authors expand the methods section to provide more clarity on potential bias in data collection and interpretation.***

**Response:** Thank you for taking the time to review our manuscript. We appreciate the elaborate feedback. ***Major Comments***

***Comment 1:
1. The potential bias with calculating a STI incidence rate have not been adequately addressed in the manuscript. This is notoriously difficult to measure, because it is often difficult to establish whether treatment was actually taken (here it appears to be self-reported), and STIs are often persistent rather than recurrent (due to lack of treatment or treatment resistant STIs). For example, a main finding in this study is that baseline CT infection predicts incident CT. However, some of these infections may not have been treated adequately at baseline (and the study did not include a test of cure).***

**Response:** Thank you. Indeed, no test of cure was performed in our study and it therefore possible that infections that were now considered “incident” were actually persistent, as we also state in the Discussion.

| **Original** | **Revised** | **Section** | **Page (lines)** |
| --- | --- | --- | --- |
| Last, although adequate treatment was offered to participants with a CT or NG infection, no test of cure was performed. Therefore, it may be possible that some of the incident cases were the result. | Last, although adequate treatment was offered to participants with a CT or NG infection, no test of cure was performed. Therefore, it may be possible that some of the incident cases were the result of treatment failure or because the participant did not take the treatment. | Discussion | 22 (321-324) |

To exclude a possible effect of persistent infections on the incidence rate, we now included three sensitivity analyses in which we excluded all women with a prevalent CT, NG, or CT and/or NG infection, respectively. The incidence rate of CT was lower when excluding participants with a prevalent CT infection at baseline, although still high (29.0 per 100 person-years). The incidence rates of NG and CT and/or NG did not differ much after exclusion of participants with a prevalent infection. The fact that the CT incidence rate was lower in this sensitivity analysis leaves open the question whether this was because treatment failures or cases not being treated were now excluded, or because re-infection was more common among women who previously had CT.

We have now added an explanation and the results of the sensitivity analysis in the manuscript:

| **Original** | **Revised** | **Section** | **Page (lines)** |
| --- | --- | --- | --- |
| - | In a sensitivity analysis, we assessed the incidence rates per 100 person-years of CT, NG, and CT and/or NG, and the determinants of these incident infections, among participants without a prevalent CT, NG, or CT and NG infection, respectively. | Statistical analysis, **Methods** | 9 (168-170) |
| - | In sensitivity analysis among participants without a prevalent CT infection at baseline, the CT incidence rate was lower (29.0 per 100 person-years, 95% CI 20.0-42.0) and multivariable analysis did not identify determinants of an incident CT infection. | Chlamydia, **Results** | 13 (216-219) |
| - | In sensitivity analysis, NG incidence rate was 20.2 per 100 person-years (95% CI 13.9-29.5) and multivariable analysis yielded similar results (data not shown). | Gonorrhoea, **Results** | 20 (266-267) |
| - | In sensitivity analysis, the incidence rate of CT and/or NG infections was lower (36.3 per 100-person-years, 95% CI 26.7-49.3) and having 3-4 lifetime sex partners was no longer significantly associated with an increased risk of an incident infection. | Chlamydia and/or gonorrhea, **Results** | 20-21 (278-281) |
| - | In sensitivity analyses among participants without prevalent infections at baseline, we showed that the incidence rate of CT, NG and CT and/or NG was still very high, although the CT incidence rate was somewhat lower than in the main analysis. The fact that the CT incidence rate was lower may be because participants with treatment failure or who did not take their treatment were now excluded, or because re-infection was more common among women with a previous CT infection. | **Discussion** | 22-23 (324-329) |

***Comment 2:
2. The process of providing results to participants is poorly described. How long after the test were participants informed. How many were reached, and how many accepted/ refused treatment? Furthermore, was treatment offered to partners, or contact cards distributed?***

**Response:** The results for Chlamydia and NG were reported in batches to the study team. In cases with positive test results, participants were informed to attend the clinic at their earliest convenience for a script for syndromic treatment. This was dispensed by the local government clinic where the study office was situated. Most cases received a script for treatment but compliance with or acceptance of treatment was not captured.

| **Original** | **Revised** | **Section** | **Page (lines)** |
| --- | --- | --- | --- |
| Participants who tested positive for HIV, CT or NG during follow-up were contacted and provided with STI results and offered treatment. | Participants who tested positive for HIV, CT or NG during follow-up were contacted, provided with STI results and offered to collect treatment at their earliest convenience. Treatment was dispensed by the local government clinic where also the study office was located. Acceptance and compliance to treatment was not captured. Sexual partners of the participant were not tested for STIs or notified of exposure to STIs during the course of this study. | Study procedure, **Methods** | 6-7 (112-117) |

***Comment 3:
3. The issue of using reusable urine cups in 102 participants, and then retesting those participants after 2 months is not clear at all. The authors state that the results were the same, but how is this possible? Were these participants not treated at baseline? Or did they start the analysis at 2-month instead of baseline for these participants? This is a potential bias in the results and is only superficially described in the methods section. In the results section (NG section) the authors then provide a p-value for the comparison, but the number and effect size is not clear.***

**Response:** Reusable urine cups were used for the first 102 participants, which were thoroughly cleaned after every use. As explained in the Methods section, we saw a very high prevalence of CT and NG at baseline (as reported in our baseline paper). Even though cups were cleaned according to the study protocol, we wanted to eliminate the possibility of cross-contamination as a reason for this high prevalence. In order to do so, we collected additional urine samples for the first 102 participants (of whom participants diagnosed with CT and/or NG had not been treated in-between visits and were provided with treatment at this second visit) in disposable cups at the second HPV vaccination visit. The prevalence of CT and NG at the first visit (when reusable cups were used) was comparable to the prevalence of CT and NG at the second visit (at which disposable cups were used), suggesting cross-contamination was not responsible for the initially observed high prevalence. As we found no evidence for cross-contamination, we started time at risk for the first 102 participants from two weeks after receiving treatment, as we did for the other participants. We have now added more information on the retesting of the first 102 participants, please see below.

| **Original** | **Revised** | **Section** | **Page (lines)** |
| --- | --- | --- | --- |
| After a very high CT and NG prevalence was found at baseline [27] additional urine samples were collected in single use, disposable cups at month 2 for the first 102 participants in order to rule out cross contamination. | After a very high CT and NG prevalence was found at baseline [27] additional urine samples were collected in single use, disposable cups at month 2 (during which those diagnosed with CT and/or NG picked up of treatment for their infection) for the first 102 participants in order to rule out cross contamination. | Study procedures, **Methods** | 6 (109-112) |
| Prevalence at baseline and month 2 for the first 102 participants was compared using Pearson’s chi square test. | In order to rule out possible cross-contamination due to the use of re-usable cups (although thoroughly cleaned), we compared the prevalence at baseline and month 2 for the first 102 participants using Pearson’s chi square test. If no evidence for cross-contamination was found, all these participants were included from their first study visit. If there was evidence for cross-contamination, all these participants would only be included from their second study visit (when the disposable cups were used). | Statistical analysis, **Methods** | 8 (140-146) |
| CT prevalence did not differ between enrolment and month 2 among women who were screened at both study visits (p=0.59, data not shown); all visits of these women were thus included in the incidence analysis. | CT prevalence did not differ between enrolment (33.3%) and month 2 (29.2%) among women who were screened at both study visits (p=0.59); therefore all visits of these women were thus included in the incidence analysis. | Chlamydia, **Results** | 13 (209-212) |
| NG prevalence did not differ between enrolment and month 2 among women who were screened for NG at those study visits (p=0.41, data not shown); all visits of these women were included in the incidence analysis. | NG prevalence did not differ significantly between enrolment (8.3%) and month 2 (12.5%) among women who were screened for NG at both these study visits (p=0.41); therefore all visits of these women were included in the incidence analysis. | Gonorrhoea, **Resuls** | 20 (258-261) |

***Comment 4:
4. How was the questionnaire administered and demographic and behavioural data gathered? What were the potential bias with the administration of the questionnaire? Please add this to the methods section. It is briefly mentioned in the limitation section of the discussion, but not in the methods section.***

**Response:** All questionnaires were tablet-based and offered in three languages (English, Xhosa and Afrikaans). Questionnaires were computer-assisted and were completed by the respondents themselves without additional help. We elaborated on this in the Methods, Study procedures:

| **Original** | **Revised** | **Section** | **Page (lines)** |
| --- | --- | --- | --- |
| At enrolment and the 7-month visit, participants completed a tablet-based questionnaire on sexual history, and socio-demographic characteristics. The questionnaire was a computer-assisted self-interview and was available in English, Xhosa, and Afrikaans. | At enrolment and the 7-month visit, participants completed a tablet-based questionnaire on sexual history, and socio-demographic characteristics (S1, S2, S3, S4, S5, and S6 File). To minimize social desirability bias, the questionnaire was a computer-assisted self-interview (instead of interviewer-administered) and was available in English, Xhosa, and Afrikaans. | Study procedure, **Methods** | 7 (123-127) |

***Comment 5:
5. There is very little information about partners, partner notification services and partner therapy uptake. Also, the conclusion should probably highlight this as a gap in the STI care cascade.***

**Response:** During the course of our study we did not test nor notify the sexual partners of our participants. We agree that this is an important gap in current STI care and we have elaborated on this further in the Conclusion. We have now made the following changes to the Methods and Conclusion:

| **Original** | **Revised** | **Section** | **Page (lines)** |
| --- | --- | --- | --- |
| - | Sexual partners of the participant were not tested for STIs or notified of exposure to STIs during the course of this study. | Study procedure, **Methods** | 7 (116-117) |
| Moreover, they indicate a need for screening and treatment programs to increase sexual health in this region. | Moreover, given the high STI prevalence in the general population of South Africa, they indicate a need for gender neutral screening and treatment programs to increase sexual health in this region. | **Conclusion** | 23 (336-338) |

***Comment 6:***

***6. Supplementary Figure 1, the flow diagram, is key to understanding the study, and should be included in the main text of the study instead of supplementary materials.***

**Response:** As per the reviewers’ suggestion, we have now included the flow diagram in the main manuscript as Fig 1.

***Minor Comments
Abstract***

***Comment 7:
l.43 suggest to add laboratory assay name to abstract***

**Response:** We have now added this information to the Method section of the Abstract.

| **Original** | **Revised** | **Section** | **Page (lines)** |
| --- | --- | --- | --- |
| At enrolment and month 6 participants were screened for CT and NG. | At enrolment and month 6 participants were screened for CT and NG (Anyplex CT/NG real-time detection method). | **Abstract** | 3 (38-39) |

***Comment 8:
l.58 suggest say ‘the majority of’ rather than ‘all’ women. For example, some may not have a partner or have no sex.***

**Response:** We agree that ‘the majority of women’ is more suited here. We made the following changes to the Conclusion of the Abstract:

| **Original** | **Revised** | **Section** | **Page (lines)** |
| --- | --- | --- | --- |
| Besides being previously infected and a higher lifetime number of sex partners, no other risk factors were found for CT and NG, suggesting that all these women were at risk. | Besides being previously infected and a higher lifetime number of sex partners, no other risk factors were found for CT and NG, suggesting that the majority of these women were at risk. | **Abstract** | 3 (52-54) |

***Introduction***

***Comment 9:
l.72 suggest change organism names to italics.***

**Response:** Thank you, we have now used italics for the name of *C. Trachomatis* and *N. gonorrhoeae* throughout the manuscript*.*

***Methods***

***Comment 10:
l.111 – 145 expand on how reusable cups were cleaned, and how the authors went about evaluating the validity of these results. How exactly did the comparison with the 2-month visit samples take place, and what were the findings?***

**Response:** The reusable cups were thoroughly cleaned using cidex immersion for at least 15 minutes and then rinsed with tap water. We elaborated on the comparison between the prevalence results under comment 3.

| **Original** | **Revised** | **Section** | **Page (lines)** |
| --- | --- | --- | --- |
| Urine cups were thoroughly cleaned after every use. | Urine cups were thoroughly cleaned after every use using cidex immersion for at least 15 minutes, after which cups were rinsed with tap water. | Study procedures**, Methods** | 6 (107-108) |

***Comment 11:
l.116 How many participants refused treatment?***

**Response:** This data is not available. Participants were given a script for the clinic pharmacy, but the number of scripts dispensed and compliance with treatment was not captured.

***Comment 12:
l.133 Where was the Anyplex stationed? In a central laboratory? What were the results turnaround times?***

**Response:** In a central laboratory at an academic hospital in the same district (Tygerberg). The turnaround time was variable due to batching of samples but was generally not more than 8 weeks.

| **Original** | **Revised** | **Section** | **Page (lines)** |
| --- | --- | --- | --- |
| - | All laboratory analyses were performed in a central laboratory at the Tygerberg hospital (Cape Town, South Africa). | Laboratory analyses**, Methods** | 7 (135-136) |

***Comment 13:
Results
The questionnaire results should be presented with more caution. For example, line 168, suggest to add ‘reported’ to receive education…***

**Response:** As the questionnaires were based on self-report, we acknowledge that the presentation of the results of the questionnaire should reflect this. We have made changed accordingly to the Result section.

| **Original** | **Revised** |  | **Page (lines)** |
| --- | --- | --- | --- |
| The majority of women included at baseline received education up to grade 12 or less (n=212, 60.4%), were single (n=334, 95.2%), and did not use birth control (n=184, 52.4%). Median age of sexual debut was 17 years [IQR 16-18] and median number of lifetime sexual partners was 2 [IQR 1-3]. | Median age at baseline was 20 years [interquartile range (IQR) 19-22] (Table 1). The majority of women included at baseline reported that they had received education up to grade 12 or less (n=212, 60.4%), were single (n=334, 95.2%), and did not use birth control (n=184, 52.4%). Median reported age of sexual debut was 17 years [IQR 16-18] and median reported number of lifetime sexual partners was 2 [IQR 1-3]. Thirteen women (3.7%) at baseline indicated that they had ever received presents, money or drugs in exchange for sex. | **Results** | 9 (180-185) |

***Comment 14:
l.191 Not clear whether these women were not treated at baseline (see major comment).***

**Response:** Participants collected their treatment at the month 2 visit and where thus not treated in between visits. Please see our response to comment 3.

***Comment 15:
l.229 The authors present a p-value, saying that there was no difference between baseline and 2-months. There should be an effect size, and further explanation to clarify this to the reader. It almost seems like the authors are confused by the retesting of the 102 participants themselves.***

**Response:** We have now added the prevalence at enrolment and month 2 for the participants who were first tested with a reusable cup. Please see our response to comment 3.

***Discussion***

***Comment 16:
l.260 As mentioned above, several of these infections may not have been cleared in the first place.***

**Response:** Please see our response to comment 1.

***Comment 17:
l.266 Suggest adding the need for better partner services and screening.***

**Response:** Thank you. We agree that partner services and gender neutral screening is very important, especially when the STI prevalence is high in the general population. We already recommended this in the Discussion to include both genders in STI screening programs. We now further elaborated on this in the Conclusion.

| **Original** | **Revised** | **Section** | **Page (lines)** |
| --- | --- | --- | --- |
| As the STI prevalence in the general population of South Africa is high [7] and in the analysis we only included participants who received adequate treatment for their CT infection, it appears that repeated CT infections were common. Given that, even with a median of two lifetime sex partners, these women were at substantial risk for incident and repeated STI infections, suggesting the need to include both genders in STI screening programs in an effort to decrease STI risk. |  | **Discussion** | 21 (296-302) |
| Moreover, they indicate a need for screening and treatment programs to increase sexual health in this region. | Moreover, given the high STI prevalence in the general population of South Africa, they indicate a need for gender neutral screening and treatment programs to increase sexual health in this region. | **Conclusion** | 23 (336-338) |

***Comment 18:
L.275 I agree with the authors that the overwhelming risk factor in this setting is young age.***

**Response:** Thank you.

***Comment 19:
l.284 ‘Tablet based questionnaires’ – this should be added to the methods as part of the expansion on the information on the questionnaire implementation.***

**Response:** The questionnaires being tablet based was already previously stated in the Methods section. Please also see our response to comment 4.

***Comment 20:
l.288 ‘treatment failure’ or person not taking the treatment in the first place?***

**Response:** Thank you, we agree that indeed incident cases could have been the results of both treatment failure and not taking the treatment. We have now added this to the Discussion.

| **Original** | **Revised** |  | **Page (lines)** |
| --- | --- | --- | --- |
| Therefore, it may be possible that some of the incident cases were the result of treatment failure. | Therefore, it may be possible that some of the incident cases were the result of treatment failure or because the participant did not take the treatment | **Discussion** | 22 (322-324) |

***Conclusion***

***Comment 21:
L.293 suggest adding ‘the majority’ instead of ‘all’***

**Response:** We revised this accordingly in both the Discussion and the Conclusion.

| **Original** | **Revised** | **Section** | **Page (lines)** |
| --- | --- | --- | --- |
| Having a prevalent CT infection at baseline and more than three lifetime sex partners were the only determinants associated with an increased risk of CT or NG, suggesting that all these young women were at high risk for an incident infection. | Having a prevalent CT infection at baseline and more than three lifetime sex partners were the only determinants associated with an increased risk of CT or NG, suggesting that the majority of these young women were at high risk for an incident infection. | **Discussion** | 21 (288-291) |
| Besides being previously infected with CT and a higher lifetime number of sex partners, no other risk factors were found for CT and NG, suggesting that all these women were at high risk for both STIs. | Besides being previously infected with CT and a higher lifetime number of sex partners, no other risk factors were found for CT and NG, suggesting that the majority of these women were at high risk for both STIs. | **Conclusion** | 23 (332-335) |

***Reviewer #2:***

***Comment 1:***

***The paper has some interesting points.***

**Response:** We are very grateful to the Reviewer for taking the time to read our manuscript and we thank the Reviewer for the compliment.

***Comment 2:***

***Can you please include a definition for incident STI infections.***

**Response:** We have now included a definition for an incident CT or NG infection in the Statistical Analysis of the Methods.

| **Original** | **Revised** | **Section** | **Page (lines)** |
| --- | --- | --- | --- |
| - | We defined an incident infection as the first positive CT and/or NG diagnosis after a negative CT and/or NG diagnoses or collection of adequate treatment. | Statistical analysis, **Methods** | 8 (157-159) |

***Comment 3:***

***There was no mention of partner testing so it is unclear if these were re-infections or new infections.***

**Response:** In this study, we were unfortunately only able to test our participants and not their partners. With that in mind, we could indeed not distinguish between re-infections and new infections. However, participants did report a median lifetime number of sexual partners of 2, while STI incidence was very high. It is thus likely that some of these incident events were re-infections, especially given that the STI prevalence in the general population of South Africa is high. We also stated this in the Discussion and this is why we advocate for gender neutral STI screening programs. Although we agree that it would have been very interesting to assess the rate of re-infection within this population as an additional research question, this would not affect the number of incident events as a re-infection is still a new infection regardless of sexual partner.

We now included a statement in the Methods section that sexual partners were not tested for STIs nor notified for potential exposure during the course of our study.

| **Original** | **Revised** | **Section** | **Page (lines)** |
| --- | --- | --- | --- |
| - | Sexual partners of the participant were not tested for STIs or notified of exposure to STIs during the course of this study. | Study procedure, **Methods** | 7 (116-117) |
| Although only few lifetime sex partners (median of 2) were reported, STI incidence was high. In addition, a prevalent CT infection at baseline increased the risk 5-fold for an incident CT infection, similar to what was observed in another South African study [23]. As the STI prevalence in the general population of South Africa is high [7] and in the analysis we only included participants who received adequate treatment for their CT infection, it appears that repeated CT infections were common. Given that, even with a median of two lifetime sex partners, these women were at substantial risk for incident and repeated STI infections, suggesting the need to include both genders in STI screening programs in an effort to decrease STI risk. | - | **Discussion** | 21 (294-302) |

***Comment 4:***

***There is no data on previous pregnancies***

**Response:** In Table 1 we state the number of women who reported a previous pregnancy (i.e. 173 women reported that they had ever been pregnant).
