# Supplementary material for: Incidence and risk factors of C. trachomatis and N. gonorrhoeae among young women from the Western Cape, South Africa: The EVRI study
Source: PLoS One. 2021 May 3;16(5):e0250871. doi: 10.1371/journal.pone.0250871 (PMC8092667; doi:10.1371/journal.pone.0250871)
Supplement: Attachment — Submitted filename: Responses to reviewers_rebuttal 2.docx [file pone.0250871.s009.docx]

**Reviewers' comments:

Comment 1:**

***I thought this was an interesting analysis that is worthwhile. As the authors note, these data arise from a trial and it's hard to grasp whether these data can be representative of a wider population. Usually, the answer with trials is no. I feel the authors used appropriate language and noted this in the limitations.

I reviewed the design and methods. Overall, this looked good, but I had a few comments below for the authors with the aim of trying to improve the statistical methods.***

**Response:** Thank you for taking the time to review the design and methods of this study and for the kind words.

**Comment 2:**
***(lines 147-148) I didn't completely understand why participants lost to follow up were excluded. If you are doing a person-time calculation, then you could include everyone for the amount of time for which data are available.***

**Response:** Testing for chlamydia and gonorrhea was done at enrolment and 6 months after the enrolment visit (line 105-106). The first 102 participants were also tested for chlamydia and gonorrhea at the month 2 visit to rule out cross contamination (line 109-112). Thus, the majority of participants had only 2 possible options for chlamydia and gonorrhea testing (enrolment and month 6). We agree that by doing a person-time calculation you can usually include data until the participant becomes lost-to-follow-up. However, if a participant missed the month 6 follow-up visit, no person-time could be included as there were only two visits.

**Comment 3: *(lines 149-151) With the uncertainty of the infection time, an interval censored survival analysis could be implemented. That type of model is specifically designed for such situations and could potentially better handle the lost to follow up.***

**Response:** Thank you for this suggestion and please also see our response to comment 2. As we had a short follow-up (6 months) and only limited visits (2 for the majority of participants), we could not take person-time into account for women who were lost-to-follow-up. Given the short interval time between STI tests, we feel the midpoint assumption gives a fairly unbiased estimation of the timing of the incident events.

**Comment 4: *(lines 162-164) If you don't utilize my prior comment, my understanding is that you've modeled the rates of events per 100 person-years. I suggest trying to model the number of incident infections instead of the rates with the person years as an offset. This is a more natural way to model this since the rates can sometimes follow some very strange distributions and have odd outliers. I think you can stick with the Poisson distribution if you like because I'm guessing the outcome will still be zeros and ones. Often, because the mean and variance are the same parameter in Poisson, that can be a very poor assumption in regression models and switching to quasipossion or negative binomial model is a better option. Though, I don't think you need that here, though I would also try to include a robust variance estimate to make sure the confidence intervals are appropriate***

**Response:** We indeed modelled the number of incident events with the person-time as offset in a Poisson model, not the incidence rates. We made this more clear now in the *Statistical Analysis*.

| **Original** | **Revised** | **Section** | **Page (lines)** |
| --- | --- | --- | --- |
| Incidence rate ratios (IRR) and 95% confidence intervals (CI) of determinants of incident CT, NG and CT and/or NG infections were assessed using a Poisson regression model. | Incidence rate ratios (IRR) and 95% confidence intervals (CI) of determinants of incident CT, NG and CT and/or NG infections were assessed using a Poisson regression model, using the number of incident infections as the outcome. | Methods | 8-9 (162-164) |

**Comment 5: *(lines 164-167) I have a few comments about the variable selection procedures utilized here. Were those variables with p < 0.20 in unvariable models the ones that are forced into the model? Otherwise, it's unclear why both bivariate screening (lines 164-5) and backward selection (lines 166-7) are used. Though, the larger problem is, with the sample sizes you have, stepwise variable selection procedures (backward included) usually do not do a good job of finding the most appropriate model (e.g.,*** [***https://doi.org/10.1002/sim.3943***](https://doi.org/10.1002/sim.3943)***). Stepwise procedures and any p-value based selection have quite a bit of evidence suggesting that they are poor at selecting the appropriate variables. For a decent summary, see the link above. Generally, it's better to select based on more robust criteria, especially measures which assess the fit of the model, such as BIC, or, better yet, a shrinkage-based estimator such as lasso or lars.***

**Response:** Our approach was as follows: we forced age at enrolment, age at first sex act and lifetime number of sex partners into the multivariable model, based on their previously found association with STI incidence. Other variables were evaluated in univariable analysis and those associated at p<0.20 were also included in the first multivariable model. We then tried to drop other variables, and examined whether dropping them would significantly improve the fit of the model, using the likelihood-ratio test (LRT). Variables whose exclusion led to a significantly worse fit (based on the LRT) were retained. So far, not much is known about the determinants for STI incidence within this younger population of women. We therefore chose to use this approach of forcing known variables into the model and including other variables (entry based on p of univariable analysis and exit based upon LRT), instead of making a theory based model (e.g. with a DAG).

We acknowledge our approach was not made sufficiently clear in the *Statistical analysis*, and we have now made the following changes.

| **Original** | **Revised** | **Section** | **Page (lines)** |
| --- | --- | --- | --- |
| Backwards selection was performed to obtain the multivariable model with the best fit. | Backwards selection, using the likelihood-ratio test, was performed to obtain the multivariable model with the best fit. | Methods | 9 (167-168) |

**Comment 6: *(Table 1) Given the sporadic missing data, did you consider using multiple imputation or some other method to try to handle the missing data?***

**Response:** Nine incident chlamydia events and 7 incident gonorrhea events were not taken into account due to missing data . Due to this limited number of missing values, a complete case analysis seemed justified.

**Comment 7: *(line 267) Since there is no word count and electronic supplements are acceptable, there is really no reason for analyses to not be reported that are important enough to be mentioned in the manuscript. Please include these analyses somewhere.***

**Response:** We agree. We have now included the results of the sensitivity analysis as Supplementary Table 1.
